# Hybrids of 1,4-Quinone with Quinoline Derivatives: Synthesis, Biological Activity, and Molecular Docking with DT-Diaphorase (NQO1)

**DOI:** 10.3390/molecules27196206

**Published:** 2022-09-21

**Authors:** Monika Kadela-Tomanek, Maria Jastrzębska, Elwira Chrobak, Ewa Bębenek, Małgorzata Latocha

**Affiliations:** 1 Department of Organic Chemistry, Faculty of Pharmaceutical Sciences in Sosnowiec, Medical University of Silesia, Katowice, 4 Jagiellońska Str., 41-200 Sosnowiec, Poland; 2 Silesian Center for Education and Interdisciplinary Research, University of Silesia, Institute of Physics, 1a Pułku Piechoty 75 Str., 41-500 Chorzów, Poland; 3 Department of Cell Biology, Faculty of Pharmaceutical Sciences in Sosnowiec, Medical University of Silesia, Katowice, 8 Jedności Str., 41-200 Sosnowiec, Poland

**Keywords:** 1,4-quinone, anticancer activity, molecular docking

## Abstract

Hybrids 1,4-quinone with quinoline were obtained by connecting two active structures through an oxygen atom. This strategy allows to obtain new compounds with a high biological activity and suitable bioavailability. Newly synthesized compounds were characterized by various spectroscopic methods. The enzymatic assay used showed that these compounds were a suitable DT-diaphorase (NQO1) substrates as evidenced by increasing enzymatic conversion rates relative to that of streptonigrin. Hybrids were tested in vitro against a panel of human cell lines including melanoma, breast, and lung cancers. They showed also a high cytotoxic activity depending on the type of 1,4-quinone moiety and the applied tumor cell lines. It was found that cytotoxic activity of the studied hybrids was increasing against the cell lines with higher NQO1 protein level, such as breast (MCF-7 and T47D) and lung (A549) cancers. Selected hybrids were tested for the transcriptional activity of the gene encoding a proliferation marker (H3 histone), cell cycle regulators (p53 and p21) and the apoptosis pathway (BCL-2 and BAX). The molecular docking was used to examine the probable interaction between the hybrids and NQO1 protein.

## 1. Introduction

Compounds containing a quinone fragment can be isolated from plants, fungi, and bacteria. Natural substances containing such moiety may have a wide spectrum of biological activities, such as: antimalarial, anticancer, antibacterial, antifungal, antiviral, and antileishmanial. The high biological activity of this group of compounds results from the physicochemical properties of the quinoline moiety, which is a heterocyclic system containing fused benzene and pyridine rings. The pyridine moiety interacts with nucleophilic and electrophilic substituent while the benzene ring creates the hydrophobic interaction. This specific chemical structure allows interaction with many biological targets, such as topoisomerase, protein kinase, telomerase, tubulin, BCl-2 family protein, protein phosphatase Cdc25, oncogenic Ras, thymidylate synthase, the matrix metalloproteinase, and mitotic kinesin-5 [1,2,3,4,5,6,7].

**Figure 1:**
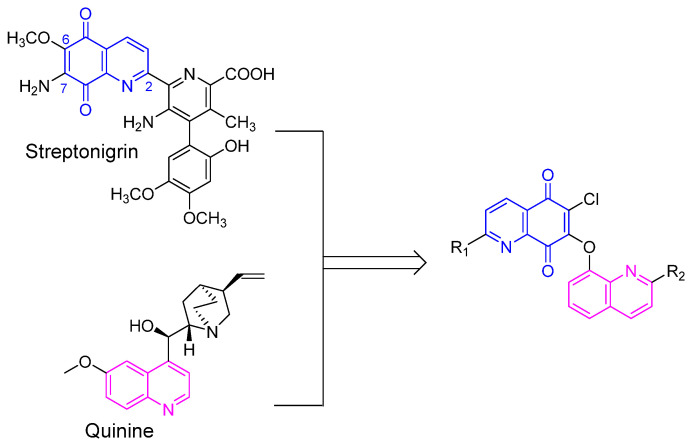
The design of title hybrids 5,8-quinolinedione (blue) connected by an oxygen atom with a quinoline (pink) moiety.

Some of the first compounds with quinoline moiety to be described were quinine and quinidine, which were isolated from cinchona bark (Figure 1). Quinine has been used in malaria treatment, whereas the quinidine exhibits antiarrhythmic properties. The quinoline scaffold is present in many natural and synthetic drugs and plays a major role in their biological activity. For example, chloroquine and mefloquine are used in antimalarial therapy, topotecan in cancer chemotherapy, and ciprofloxacin in bacterial infections [8,9,10,11].

Quinoline compounds constitute a large group of substances, which are classified according to their structural modifications [5]. For example, natural, such as streptonigrin, and synthetic 5,8-quinolinedione compounds show wide spectrum of biological activities such as anticancer, antibacterial, and antiviral. The activity depends on the substituent at the positions C-6, C-7 and C-2 of the 5,8-quinolinedione moiety (Figure 1) [12,13,14,15,16].

Studies on the mechanism of their biological activity showed that they activate the enzyme DT-diaphorase (NQO1), which is a flavoprotein enzyme localized in cytosol (>90%), mitochondria, endoplasmic reticulum and nucleus [17,18,19,20]. The high level of NQO1 protein was established in many human solid tumors found in breast, lung, colon and brain [21,22]. The NQO1 enzyme catalyzes the reduction in the quinone moiety to a hydroquinone using the NADH or NADPH as cofactors. As a side product, reactive oxygen species (ROS) are formed, which cause mutation of DNA [23,24,25].

In this paper, we describe a new hybrid compounds containing 1,4-quinone and quinoline moieties connected by an oxygen atom (Figure 1). We assume, that the combination of two active moieties may result in the increased activity and better bioavailability of resulting product. New hybrids were tested as substrate for the NQO1 protein. Their anticancer activities were assessed against a six human cancer cell lines. For the selected compounds the molecular mechanism of activity was evaluated. Furthermore, the molecular docking study was used to examine the interaction between hybrids and the NQO1 enzyme.

## 2. Results and Discussion

### 2.1. Chemistry

**Scheme 1:**
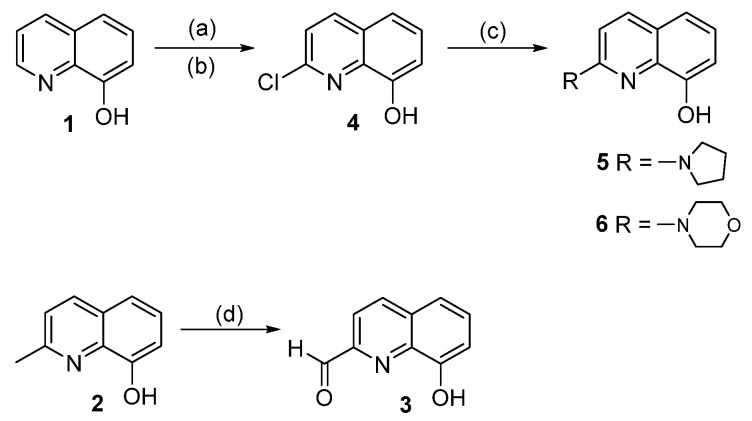
Synthesis route of compounds **3**–**6**. Reagents: (a) mCBPA, CH_2_Cl_2_, rt, 12 h; (b) PPh_3_, Cl_3_CCN, toluene, reflux, 4 h; (c); amine, MW, 110 °C, 10 min; (d) SeO_2_, dioxane, reflux, 24 h.

The general pathway for preparing the 2-substituted derivatives **3**–**6** from commercially available quinolin-8-ol **1** and 2-methyl-8-hydroxyquinoline **2** is presented in Scheme 1. The 2-amine derivatives **5**–**6** were obtained in a multistep procedure. First, compound **1** was converted to 2-chloro-8-hydroxyquinoline according to the literature procedure [26]. The target molecules **5**–**6** were obtained in the condensation reaction between compound **4** and pyrrolidine or morpholine. The quinoline-2-carbaldehyde **3** was obtained by oxidation of 2-methyl-8-hydroxyquinoline **2** with selenium (IV) oxide [27]. The chemical structures of compounds **3**–**6** were confirmed by spectroscopic (^1^H and ^13^C NMR) methods.

In our previous research, we described the effective method for the synthesis of 5,8-quinolinedione connected to the alkoxyl, alkenyl, enediyne and betulin subunits [14,15,28,29]. This method was used to synthesize hybrid 1,4-quinone with quinoline derivatives. As substrates three different dichloro derivatives of 5,8-quinolinedione (**7**–**9**) and 1,4-naphthoquinone (**10**) were used.

**Scheme 2:**
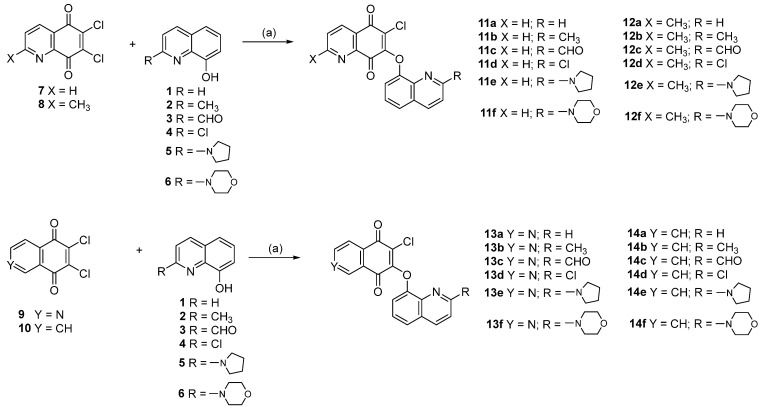
Synthesis route of hybrids **11**–**14**. Reagents: (a) K_2_CO_3_, THF, rt, 24 h.

As seen in Scheme 2, compounds **1**–**6** were connected to 1,4-quinone derivatives **7**–**10** in the reaction with potassium carbonate and tetrahydrofuran (THF).

After purification, hybrids **11**–**14** were obtained with a yield of 47–94%. The structures of all new compounds **11**–**14** were confirmed by ^1^H NMR, ^13^C NMR, and HR-MS spectroscopic methods.

### 2.2. Biological Activity

#### 2.2.1. Enzymatic Assay

**Figure 2:**
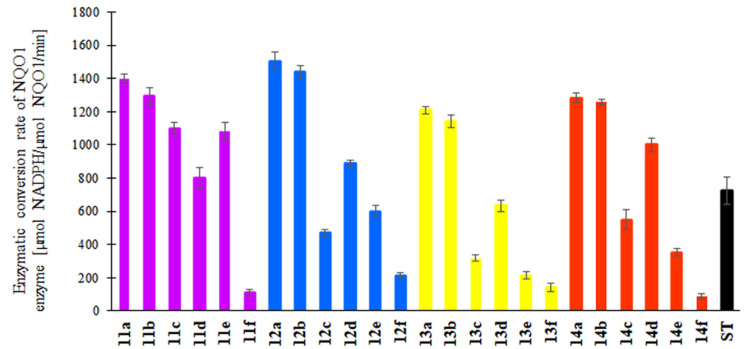
Enzymatic conversion rate of NQO1 of hybrids **11**–**14** and streptonigrin (**ST**) monitored at the absorbance of A340 nm.

The interaction between 1,4-quinone moiety of hybrid and NQO1 protein leads to reduction in quinone to hydroquinone and production of reactive oxide form (ROS) [30,31]. According to a literature method, the ability of hybrids **11**–**14** and streptonigrin (**ST**) to be a substrate for NQO1 was examined by measuring the oxidation of NADPH to NADP^+^ [29,32]. The results are presented in Figure 2 and in Table S1.

Most of the tested compounds show higher enzymatic conversion rate of NQO1 than streptonigrin (**ST**) (725 μmol NADPH/μmol NQO1/min), indicating that they are suitable substrates for NQO1.

In the group of 5,8-quinolinedione compounds **11a**–**f**, the **11a** exhibits the highest enzymatic conversion rate, which is about 2-times higher than streptonigrin. Introduction of methyl group at the C-2 position of the 5,8-quinolinedione moiety (**12a**–**f**) has a negligible influence on conversion rates, except **11c** and **11e**, which are 2.5-and 1.8-times more active than **12c** and **12e**, respectively. Comparing the activities of 5,8-quinolinedione compounds (**11a**–**f**) and 5,8-isoquinolinedione hybrids (**13a**–**f**) shows that replacement of nitrogen atom from N-1 to N-2 position decreases the enzymatic conversion rates. The same correlation is observed for 5,8-quinolinedione (**11a**–**f**) and 1,4-naphthoquinone (**14a**–**f**) derivatives. In the group of hybrids with quinolin-8-yloxy and 2-methylquinolin-8-yloxy substituents (**11a**–**b**, **12a**–**b**, **13a**–**b** and **14a**–**b**), the NQO1 activity depends on the type of 1,4-quinone moiety and the order is as follows: 2-methyl-5,8-quinolinedione > 5,8-quinolinedione > 1,4-naphthoquinone > 5,8-isoquinolinedione.

It has been found that the substituent at the C-2 position of quinoline moiety influences the enzymatic conversion rates. Compounds with hydrogen atom (**11a**, **12a**, **13a** and **14a**) and methyl group (**11b**, **12b**, **13b** and **14b**) at this position exhibit comparable activities. The oxidation of methyl (**11b**, **12b**, **13b,** and **14b**) to carboxy (**11c**, **12c**, **13c,** and **14c**) group decreases the conversion rates. Replacement of chloride atom (**11d**, **12d**, **13d,** and **14d**) with amine substituent (**11e**–**f**, **12e**–**f**, **13e**–**f,** and **14e**–**f**) leads also to reduction in NQO1 activity.

#### 2.2.2. Anticancer Activity

Anticancer activity of compounds **11**–**14** was designated against following human cancer cell lines: melanoma (Colo-829), ovarian cancer (SK-OV-3), breast cancer (MDA-MB-231, T47D and MCF-7), lung cancer (A549) and normal fibroblasts (HFF-1). As a reference substance, the doxorubicin was used. The results are presented in Table 1 as IC_50_, where IC_50_ means the concentration of tested compounds (µM) causing the growth inhibition of 50% for given tumor cells. The negative value (Neg.) means that the IC_50_ is higher than 30 µM. Selectivity index (SI) as the ratio of the IC_50_ for normal fibroblast (HFF-1) to IC_50_ of the corresponding cell line, is presented in Table S2.

Most of the tested hybrids show lower activity than doxorubicin against all cancer cell lines. In the series of tested compounds **11**–**14**, the 1,4-naphthoquinone derivatives (**14a**–**f**) show the lowest activity. Analysis of the structure-activity relationship indicates that the activity depends on the type of 1,4-quinone moiety, what means that the nitrogen atom is necessary to preserve the anticancer effect (Table 1).

As seen in Table 1, hybrids **11**–**14** exhibit the lowest activity against the melanoma cell line (Colo-829). The highest activity against the melanoma cell line is seen for the 6-chloro-7-(quinolin-8-yloxy)isoquinoline-5,8-dione (**13a**) for which the IC_50_ is equal to 0.17 µM.

Comparing the activity of 5,8-quinolinedione compounds (**11a**–**f**) against the ovary (SK-OV-3) cell line shows that the introduction of a substituent at the C-2 position of quinoline moiety (**11b**) causes an increase in activity. The replacement of a chlorine atom (**11d**) with an amine substituent (**11e**–**f**) leads to a decrease in activity.

The breast cancer cells lines used for testing have different immunoprofile classes. Two of them (T47D and MCF-7) belong to Luminal A, while MDA-MB-231 to the Claudin-low class. According to literature, the Luminal A cell line subtype is less aggressive than the Claudin-low class [33]. Most hybrids **11**–**14** exhibit higher activity against Claudin-low class cells (MDA-MB-231) than Luminal A class cell lines (T47D and MCF-7). The activity against the MDA-MB-231 cell line depends on the type of 1,4-quinone moiety and the order is as follows: 5,8-isoquinolinedione > 2-methyl-5,8-quinolinedione > 5,8-quinolinedione > 1,4-naphthoquinone. Comparing the IC_50_ value against T47D and MCF-7 shows that compounds **11**–**13** exhibit comparable activity. Important feature is observed for 1,4-naphthoquinone hybrids **14a**–**f**, which exhibit low activity against MCF-7 and no effect against T47D.

The cell lines used in the research have different expression levels of a gene encoding NQO1 protein. According to literature, the cell lines are classified into six categories depending on the level of RNA expression denoted as the normalized transcript expression values (nTPM). The A549 line belongs to the enhanced (nTPM = 2724.0) while T47D and MCF-7 belong to low specificity lines (nTPM in the range of 725.9–631.3). For the other lines the nTPM values were not detected [34,35].

From this perspective, hybrids **11**–**14** exhibit the higher activity against cancer cell line with enhanced level of gene encoding NQO1 enzyme. Most of the tested hybrids exhibit the highest activity against the A549 cell line. The exception is compound **11e**, which is almost 6-times more active against MCF-7 than A549. Therefore, the effect depends on the nTPM for each line and the order is as follows: A549 > MCF-7 > T47D.

Hybrids with 5,8-quinolinedione (**11a**–**f** and **12a**–**f**) and 5,8-isoquinolinedione (**13a**–**f**) moieties exhibit higher activity than compounds with 1,4-naphthoquinone (**14a**–**f**) against the lung cancer cell line (A549). In general, for this group of compounds, it can be seen that position of nitrogen atom in 5,8-quinolinedione moiety influences anticancer effect. Hybrids with the 5,8-isoquinolinedione moiety (**13a**–**f**) are characterized by the highest anticancer effect. For compounds **12a**–**f**, the introduction of the methyl group at the C-2 position of the 5,8-quinolinedione moiety affects their cytotoxicity showing that most of them have higher activity than compounds **11a**–**f** against lung(A549) cell line.

The second important aspect affecting the activity is the substituent at the C-2 position of the quinolinoxy moiety. Generally, a trend is that the introduction of methyl group (**11b**, **12b**, **13b** and **14b**) decreases the cytotoxicity. However, the oxidation of methyl group (**11b**, **12b**, **13b** and **14b**) to the carbonyl group (**11c**, **12c**, **13c** and **14c**) reduces the activity against the A549 line. The replacement of methyl group with chlorine atom (**11d**, **12d**, **13d** and **14d**) significantly increases activity. The amine substituent (**11e**–**f**, **12e**–**f**, **13e**–**f** and **14e**–**f**) shows little effect on activity. In the series of 5,8-isoquinolinediones (**13a**–**f**) the activity depends on a group at the C-2 position of quinolinoxy moiety and the order is as follows: hydrogen (**13a**) > chloride (**13d**) > methyl (**13b**) > carbonyl (**13c**) > morpholinyl (**13f**) > piperidin-1-yl (**13e**).

The hybrids **11**–**14** show less cytotoxicity against normal fibroblast cell line (HFF-1) than doxorubicin. However, no correlation was observed between the type of 1,4-quinone moiety and the cytotoxicity of the compound (Table 1). 

Most of tested hybrids **11**–**14** show higher selectivity index than doxorubicin (SI = 6.00) against the A549 cell line. However, the highest SI index was obtained for **13a** and **13d**, reaching values of 923.00 and 184.83, respectively.

#### 2.2.3. Apoptosis Analysis

**Figure 3:**
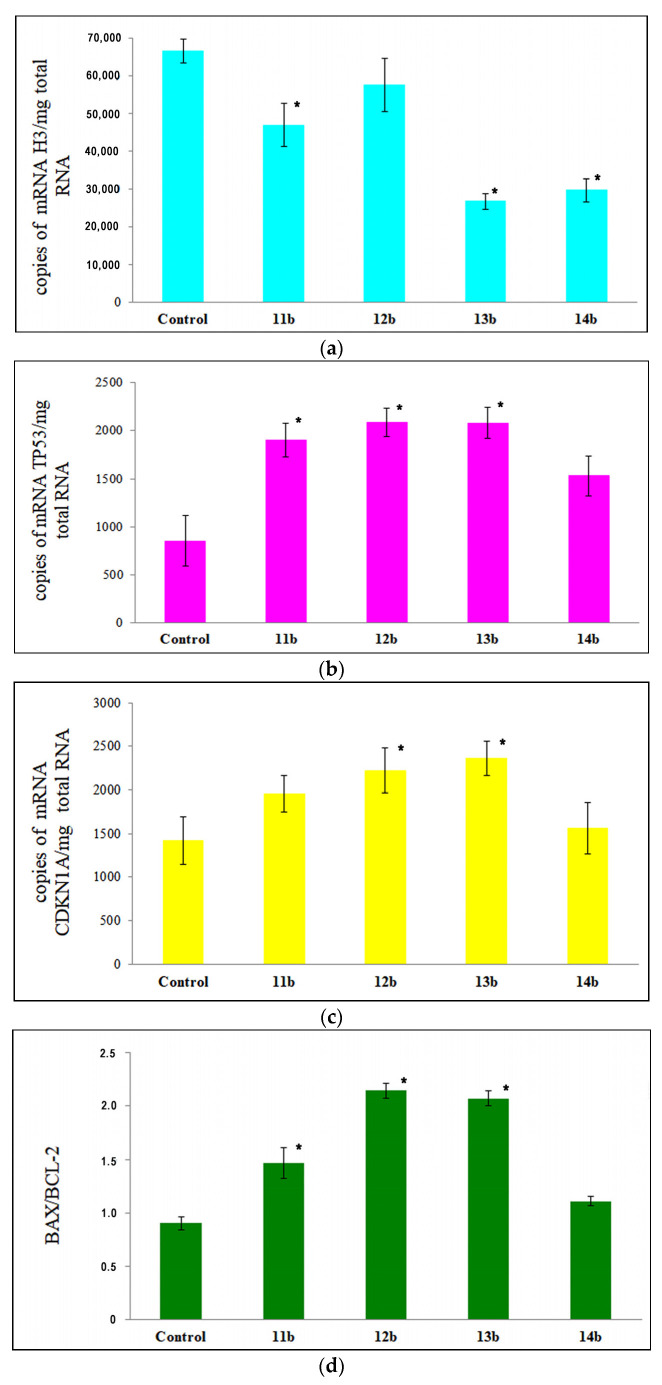
Effect of hybrids **11b**, **12b**, **13b** and **14b** on transcriptional activity of: (**a**) H3; (**b**) TP53; (**c**) CDKN1A; (**d**) BAX/BCL-2 for A549 cells. * *p* < 0.05 compared with the untreated control.

The mechanism of anticancer activity was determined by detection of transcriptional activity of the genes encoding a proliferation marker (H3 histone), cell cycle regulators (p53 and p21), and the mitochondrial apoptosis pathway (BCL-2 and BAX) (Figure 3a–d). The derivatives **11b**, **12b**, **13b**, and **14b** were selected for this study. The research was carried out after 24 h of exposure of the A549 cell line to the compounds under study (at a concentration equal to half of IC_50_).

The tested compounds cause a decrease in the number of mRNA copies of the histone H3 gene. The highest effect is observed for compounds **13b** and **14b**, which reduce the amount of mRNA copies by half. These results suggest that the activity against A549 cells could be related to the inhibition of cell proliferation (Figure 3a).

The interaction of hybrids with the NQO1 protein leads to the production of reactive oxygen species (ROS). The increased concentration of ROS can damage many biological structures such as proteins, lipids, and nucleic acids. The TP53 is a suppressor gene that prevents transmission of genetic disorders in newly formed cells [36,37]. As seen in Figure 3b, the tested hybrids generate a change in expression of the TP53. Compounds with the 5,8-quinolinedione (**11b** and **12b**) and 5,8-isoquinolinedione (**13b**) moieties show significant increase in the TP53 gene expression. However, the type of 5,8-quinolinedione moiety has no effect on the TP53 gene expression. Hybrid **14b** containing the 1,4-naphthoquinone moiety causes a slight increase in expression of this gene.

The TP35 gene encodes the p53 protein, which controls the expression of many genes involved in the apoptosis pathway. One of these genes is CDKN1A, which codes for the p21 protein involved in cell cycle arrest in the G1 phase [38,39,40]. The highest increase in copies of mRNA is observed for **13b**, whereas the **14b** causes a slight effect on expression of CDKN1A in lung cancer (A549) (Figure 3c). Summarizing, the nitrogen atom at 1,4-quinone moiety is necessary for activation of TP53 and CDKN1A genes.

The p53 protein affects the expression of the BCL-2 protein family. The BCL-2 family includes both pro-apoptotic (BAX) and anti-apoptotic (BCL-2) proteins [41]. Figure 3d shows the BAX and BCL-2 of mRNA copies for the tested compounds and the control. A comparison of the BAX/BCL-2 ratio of mRNA copies for the control and tested compounds show that hybrids activate the mitochondrial apoptosis pathway.

### 2.3. Molecular Docking Study

Enzymatic studies show that hybrids are a suitable substrate for the NQO1 enzyme. The molecular docking study was used to examine the possible interaction between hybrids (**11**–**14**) and NQO1 protein. The NQO1 protein was obtained from PDB (PDB ID:2F1O) as a complex with the flavin adenine dinucleotide (FAD) cofactor. As a reference substance, streptonigrin (**ST**) was used. The scoring function of the tested compounds and streptonigrin is presented in Table 2. The lowest scores of the binding energy (kcal/mol) of protein-ligand complexes correspond to a strong binding affinity and the most probable ligand-protein system.

Compared to streptonigrin (**ST**), hybrids show lower binding energy with NQO1, which means that these complexes are more stable. Analysis of the scoring values shows that ligands with 5,8-quinolinedione (**11a**–**f** and **12a**–**f**) and 5,8-isoquinolinedione (**13a**–**f**) moieties are bonded to the active site of the protein more strongly than in the case of 1,4-naphthoquinone (**14a**–**f**) hybrids. However, the type of 5,8-quinolinedione moiety slightly influences the scoring value. This result is consistent with the NQO1 enzymatic study.

**Figure 4:**
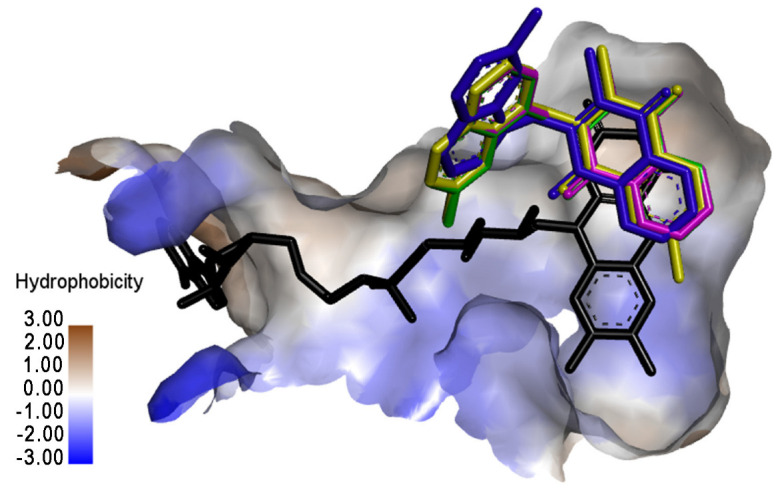
Superposition of docked ligands: **11b** (green), **12b** (yellow), **13b** (violet), **14b** (blue), and FAD cofactor (black) in the binding site of the NQO1 enzyme. Protein is colored according to its hydrophobicity scale.

As seen in Figure 4, the hybrids are localized in a hydrophobic matrix of the active enzyme site. The 1,4-quinolinedione moiety is located near the FAD cofactor and side chains of aromatic residue in positions Tyr128, Trp105, and Phe178.

**Figure 5:**
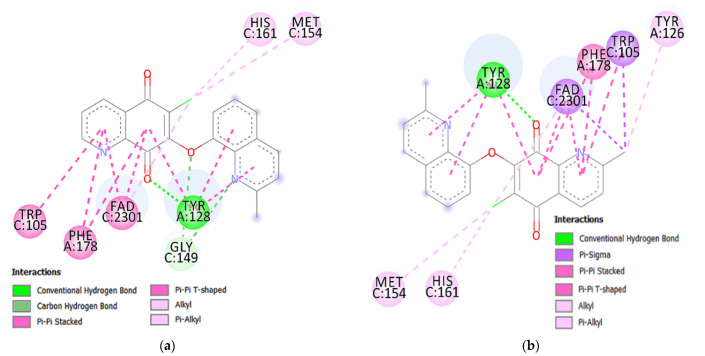

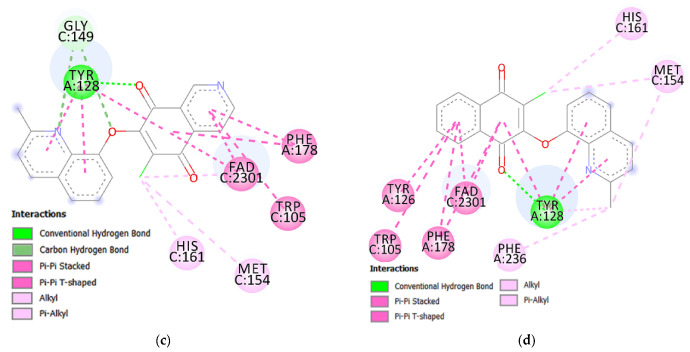
Visualization of interactions between NQO1 enzyme and hybrid: (**a**) **11b**; (**b**) **12b**; (**c**) **13b**; (**d**) **14b**.

For the selected hybrids **11b**, **12b**, **13b,** and **14b**, complete models of the interaction in 2D (Figure 5) and 3D (Figure S1) views are presented. Details of the type and length of binding between ligands and enzyme residues are summarized in Table S3.

In tested hybrids containing the 1,4-quinone moiety, the carbonyl atom creates the hydrogen bond with the hydroxy group of tyrosine (TYR128) and hydrophobic interaction with phenylalanine (PHE178), tryptophan (TRP105), histidine (HIS161), methionine (MET154) and a FAD cofactor (Figure 5a–d). Furthermore, in compounds **11b** and **13b,** the oxygen atom at the C-7 position of 5,8-quinolinedione moiety creates a weak hydrogen bond with the CH_2_ group of glycine (GLY149) (Figure 5a,c). The quinolinoxy group in ligands (**11b**, **12b**, **13b**, and **14b**) creates the hydrophobic interaction with tyrosine (TYR128) (Figure 5a–d). For hybrid **14b**, the hydrophobic interaction with phenylalanine was additionally observed (Figure 5d).

It was found that derivative **12b** shows the highest enzymatic activity among all tested compounds. This effect may be due to the hydrophobic interaction between the methyl group at the C-2 position of the 5,8-quinolinedione moiety and the FAD cofactor (Figure 5b).

## 3. Materials and Methods

### 3.1. Chemistry

The nuclear magnetic resonance (NMR) spectra were performed using the Bruker Avance 600 spectrometer (Bruker, Billerica, MA, USA) in CDCl_3_ or DMSO-d_6_ solvents. Chemical shifts (δ) are reported in ppm and *J* values in Hz. Multiplicity is designated as singlet (s), board singlet (bs), doublet (d), doublet of doublets (dd), and multiplet (m). High-resolution mass spectral analysis (HR-MS) was performed using the Bruker Impact II instrument (Bruker, Billerica, MA, USA). Calculation of the theoretical molecular mass for compounds was performed using “The Exact Mass Calculator, Single Isotope Version” (Available online: http://www.sisweb.com/referenc/tools/exactmass.htm; Ringoes, NJ, USA)) (accessed on 10 September 2022). Melting points were measured by the Electrothermal IA 9300 melting point apparatus. A microwave reactor (Discover SP-D, CEM Corporation, Matthews, NC, USA) was used in the synthesis of 2-aminoquinolin-8-ols.

The 8-hydroxyquinoline-2-carbaldehyde **3** and 2-chloroquinolin-8-ol **4** were prepared according to the method described in the literature [26,42].

The 1,4-quinones **7**–**9** were obtained according to literature methods [12,43]. The 2,3-dichloro-1,4-naphthoquinon **10** was purchased in Merck (Darmstadt, German).

Synthesis of 2-aminoquinolin-8-ols **5**–**6**

The 2-chloroquinolin-8-ol **4** (0.3 g; 1.55 mmol) was added to 3 mmol of amine (piperidine or morpholine). The reaction vessel was placed in a microwave reactor, and the reaction was carried out for 10 min at a temperature of 110 °C at a maximum wave power (300 W). After cooling, the reaction mixture was diluted with methylene chloride (10 mL) and washed with 15 mL of water. The obtained organic layer was dried with magnesium sulfate and concentrated with a vacuum evaporator. The crude product was purified by column chromatography (SiO_2_, chloroform/ethanol, 15:1, *v*/*v*) to give pure products **5**–**6**.

2-(piperidin-1-yl)quinolin-8-ol (**5**): 0.21 g (0.127 mmol); yield 60%; oil. The spectral data were confirmed by literature [44].

2-morpholinoquinolin-8-ol (**6**): 0.28 g (0.127 mmol); yield 80%; oil. The spectral data were confirmed by literature [44].

Synthesis of hybrids **11**–**14**

The 1,4-quinone compounds **7**–**10** (0.44 mmol), quinoline-8-ol derivatives **1**–**6** (0.44 mmol) and potassium carbonate (0.061 g, 0.44 mmol) were dissolved in tetrahydrofuran. After 24 h at room temperature, the reaction mixture was concentrated with a vacuum evaporator. The crude product was purified by column chromatography (SiO_2_, chloroform/ethanol, 15:1, *v*/*v*) to give pure products **11**–**14**.

6-chloro-7-(quinolin-8-yloxy)quinoline-5,8-dione (**11a**): 0.086 g (0.256 mmol); Yield 58%; mp 112–114 °C; ^1^H NMR (DMSO-d_6_, 600 MHz) δ 7.46 (d, J = 8.4 Hz, 1H), 7.59 (d, J = 8.4 Hz, 1H), 7.73 (dd, J = 4.2 Hz, J = 8.4 Hz, 1H), 7.99 (dd, J = 4.2 Hz, J = 7.8 Hz, 1H), 8.48 (dd, J = 1.8 Hz, J = 7.8 Hz, 1H), 8.53 (dd, J = 1.8 Hz, J = 7.8 Hz, 1H), 8.99 (dd, J = 1.8 Hz, J = 4.2 Hz, 1H), 9.17 (dd, J = 1.8 Hz, J = 4.8 Hz, 1H), 10.48 (bs, 1H); ^13^C NMR (DMSO-d_6_, 150 MHz) δ 117.6, 120.7, 123.3, 128.6, 129.0, 129.4, 129.5, 135.3, 136.7, 138.8, 143.8, 144.5, 147.6, 149.1, 151.3, 155.0, 176.6, 181.5; HRAPCIMS *m*/z 337.0338 (calcd for C_18_H_9_ClN_2_O_3_, 337.0379) (Figure S2).

6-chloro-7-[(2-methylquinolin-8-yl)oxy]quinoline-5,8-dione (**11b**): 0.105 (0.300 mmol); yield 68%; mp 110–112 °C; ^1^H NMR (DMSO-d_6_, 600 MHz) δ 2.74 (s, 3H), 7.32 (d, J = 8.4 Hz, 1H), 7.47 (d, J = 8.4 Hz, 1H), 7.55 (dd, J = 4.2 Hz, =8.4 Hz, 1H), 7.99 (dd, J = 4.2 Hz, J = 7.8 Hz, 1H), 8.30 (dd, J = 1.2 Hz, J = 7.8 Hz, 1H), 8.47 (dd, J = 1.8 Hz, J = 7.8 Hz, 1H), 9.12 (dd, J = 1.8 Hz, J = 4.8 Hz, 1H), 9.88 (bs, 1H); ^13^C NMR (DMSO-d_6_, 150 MHz) δ 25.2, 114.8, 117.5, 124.0, 127.7, 128.7, 129.5, 130.5, 130.8, 131.2, 135.3, 144.5, 147.6, 155.0, 157.9, 176.6, 181.5. HRAPCIMS m/z 351.0549 (calcd for C_19_H_11_ClN_2_O_3_, 351.0536) (Figure S3).

8-[(6-chloro-5,8-dioxo-5,8-dihydroquinolin-7-yl)oxy]quinoline-2-carbaldehyde (**11c**): 0.127 g (0.348 mmol); yield 79%; mp 168–170 °C; ^1^H NMR (CDCl_3_, 600 MHz) δ 7.46 (d, J = 8.4 Hz, 1H), 7.64 (d, J = 8.4 Hz, 1H), 7.82 (dd, J = 4.2 Hz, J = 8.4 Hz, 1H), 8.09 (d, J = 1.2 Hz, 1H), 8.19 (dd, J = 4.2 Hz, J = 7.8 Hz, 1H), 8.35 (dd, J = 1.2 Hz, J = 7.8 Hz, 1H), 8.56 (dd, J = 1.8 Hz, J = 7.8 Hz, 1H), 9.17 (dd, J = 1.8 Hz, J = 4.8 Hz, 1H), 10.25 (s, 1H); ^13^C NMR (CDCl_3_, 150 MHz) δ 116.4, 121.6, 126.9, 127.3, 129.6, 131.0, 137.4, 139.0, 142.5, 148.7, 158.2, 159.0, 162.9, 178.0, 181.9, 190.1; HRAPCIMS *m*/*z* 365.0351 (calcd for C_19_H_9_ClN_2_O_4_, 365.0329) (Figure S4).

6-chloro-7-[(2-chloroquinolin-8-yl)oxy]quinoline-5,8-dione (**11d**): 0.103 g (0.278 mmol); yield 63%; mp 123–125 °C; ^1^H NMR (CDCl_3_, 600 MHz) δ 7.64 (d, J = 9.0 Hz, 1H), 7.80 (m, 2H), 7.95 (dd, J = 4.2 Hz, J = 7.8 Hz, 1H), 8.33 (d, J = 8.4 Hz, 1H), 8.44 (d, J = 8.4, 1H), 8.65 (dd, J = 1.8 Hz, J = 7.8 Hz, 1H), 9.15 (dd, J = 1.8 Hz, J = 4.8 Hz, 1H); ^13^C NMR (CDCl_3_, 150 MHz) δ 111.6, 116.4, 123.0, 127.1, 128.3, 128.8, 129.8, 131.3, 135.5, 144.6, 147.5, 148.6, 155.1, 158.6, 176.4, 181.8; HRAPCIMS *m*/*z* 371.0019 (C_18_H_8_Cl_2_N_2_O_3_, 370.999) (Figure S5).

6-chloro-7-{[2-(pyrrolidin-1-yl)quinolin-8-yl]oxy}quinoline-5,8-dione (**11e**): 0.159 g (0.392 mmol); yield 89%; oil; ^1^H NMR (CDCl_3_, 600 MHz) δ 2.10 (bs, 4H), 3.67 (bs, 4H), 6.85 (d, J = 9.6 Hz, 1H), 7.05 (m, 2H), 7.27 (d, J = 8.4 Hz, 1H), 7.75 (dd, J = 4.2 Hz, J = 8.4 Hz, 1H), 7.94 (dd, J = 4.2 Hz, J = 7.8 Hz, 1H), 8.53 (dd, J = 1.8 Hz, J = 7.8 Hz, 1H), 9.11 (dd, J = 1.8 Hz, J = 4.8 Hz, 1H); ^13^C NMR (CDCl_3_, 600 MHz) δ 24.4, 45.7, 110.9, 116.8, 121.5, 127.0, 127.2, 128.0, 128.2, 134.4, 143.0, 143.9, 146.2, 153.8, 175.6, 179.9; HRAPCIMS *m*/*z* 406.0967 (calcd for C_22_H_16_ClN_3_O_3_, 406.0958) (Figure S6).

6-chloro-7-{[2-(morpholin-4-yl)quinolin-8-yl]oxy}quinoline-5,8-dione (**11f**): 0.175 g (0.414 mmol); yield 94%; oil; ^1^H NMR (DMSO-d_6_, 600 MHz) δ 3.73 (m, 4H), 3.76 (m, 4H), 7.04 (d, J = 8.4 Hz, 1H), 7.31 (dd, J = 4.2 Hz, J = 8.4 Hz, 1H), 7.94 (dd, J = 4.2 Hz, J =7.8 Hz, 1H), 8.11 (d, J = 9.0 Hz, 1H), 8.45 (dd, J = 1.8 Hz, J = 7.8 Hz, 1H), 9.11 (dd, J = 1.8 Hz, J = 4.8 Hz, 1H), 9.14 (bs, 1H); ^13^C NMR (DMSO-d_6_, 150 MHz) δ 25.1, 45.1, 111.4, 115.8, 117.3, 123.5, 123.7, 129.0, 129.5, 135.3, 137.3, 138.0, 144.3, 144.2, 147.6, 148.6, 155.0, 156.8, 176.7, 181.6; HRAPCIMS *m*/*z* 422.0941 (calcd for C_22_H_16_ClN_3_O_4_, 422.0908) (Figure S7).

6-chloro-2-methyl-7-(quinolin-8-yloxy)quinoline-5,8-dione (**12a**): 0.071 g (0.202 mmol); yield 49%; mp 115–117 °C; ^1^H NMR (DMSO-d_6_, 600 MHz) δ 2.72 (s, 3H), 7.79 (m, 2H), 8.16 (d, J = 8.4 Hz, 1H),8.33 (d, J = 8.4 Hz, 1H), 8.46 (d, J = 7.8 Hz, 1H), 8.67 (dd, J = 1.8 Hz, J = 7.8 Hz, 1H), 9.14 (dd, J = 1.8 Hz, J = 4.8 Hz, 1H), 10.38 (bs, 1H); ^13^C NMR (DMSO-d_6_, 150 MHz) δ 25.1, 115.8, 120.7, 123.6, 127.8, 128.7, 135.1, 137.6, 146.9, 155.4, 164.2, 178.7, 180.9; HRAPCIMS m/z 351.0539 (calcd. for C_19_H_11_ClN_2_O_3_, 351.0536) (Figure S8).

6-chloro-2-methyl-7-[(2-methylquinolin-8-yl)oxy]quinoline-5,8-dione (**12b**): 0.078 (0.215 mmol); yield 52%; mp 130–132 °C; ^1^H NMR (DMSO-d_6_, 600 MHz) δ 2.71 (s, 3H), 2.79 (s, 3H), 7.66 (d, J = 8.4 Hz, 1H), 7.78 (d, J = 8.4 Hz, 1H), 8.06 (d, J = 8.4 Hz, 1H), 8.20 (d, J = 8.4 Hz, 1H), 8.42 (d, J = 7.8 Hz, 1H), 8.50 (d, J = 8.4 Hz, 1H), 10.40 (bs, 1H); ^13^C NMR (DMSO-d_6_, 150 MHz) δ 25.0, 25.4, 119.6, 122.5, 123.8, 124.2, 127.5, 127.9, 128.6, 135.1, 136.0, 137.5, 148.4, 151.3, 154.6, 161.0, 173.3, 181.0; HRAPCIMS *m*/*z* 365.0699 (calcd. for C_20_H_13_ClN_2_O_3_, 365.0693) (Figure S9).

8-[(6-chloro-2-methyl-5,8-dioxo-5,8-dihydroquinolin-7-yl)oxy]quinoline-2-carbaldehyde (**12c**): 0.116 g (0.306 mmol); yield 74%; mp 184–186 °C; ^1^H NMR (DMSO-d_6_, 600 MHz) δ 2.73 (s, 3H), 7.81 (d, J = 8.4 Hz, 1H), 7.96 (d, J = 8.4 Hz, 1H), 8.23 (d, J = 8.4 Hz, 1H), 8.27 (d, J = 8.4 Hz, 1H), 8.45 (d, J = 1.8 Hz, 1H) 8.46 (d, J = 7.8 Hz, 1H), 8.90 (d, J = 8.4 Hz, 1H), 10.29 (s, 1H); ^13^C NMR (DMSO-d_6_, 150 MHz) δ 25.0, 113.2, 119.3, 123.4, 127.8, 128.0, 131.0, 135.2, 139.5, 148.4, 153.2, 162.8, 164.3, 173.3, 180.8, 193.8; HRAPCIMS *m*/*z* 379.0491 (calcd. for C_20_H_11_ClN_2_O_4_, 379.0486) (Figure S10).

6-chloro-2-methyl-7-[(2-chloroquinolin-8-yl)oxy]quinoline-5,8-dione (**12d**): 0.092 g (0.240 mmol); yield 58%; mp 120–122 °C; ^1^H NMR (CDCl_3_, 600 MHz) δ 2.86 (s, 3H), 7.63 (m, 2H), 7.94 (m, 2H), 8.32 (d, J = 8.4 Hz, 1H), 8.44 (d, J = 8.4 Hz, 1H), 8.52 (d, J = 7.8 Hz, 1H); ^13^C NMR (CDCl_3_, 150 MHz) δ 25.3, 115.0, 116.3, 121.5, 124.1, 127.6, 130.5, 135.2, 139.3, 143.3, 144.4, 151.8, 158.3, 161.1, 178.1, 180.1; HRAPCIMS *m*/*z* 385.0159 (caldc. for C_19_H_10_Cl_2_N_2_O_3_, 385.0147) (Figure S11).

6-chloro-2-methyl-7-{[2-(pyrrolidin-1-yl)quinolin-8-yl]oxy}quinoline-5,8-dione (**12e**): 0.144 g (0.343 mmol); yield 83%; oil; ^1^H NMR (DMSO-d_6_, 600 MHz) δ 1.99 (bs, 4H), 2.72 (s, 3H), 3.60 (bs, 4H), 6.97 (m, 2H), 7.25 (d, J = 8.4 Hz, 1H), 7.79 (d, J = 8.4 Hz, 1H), 8.04 (d, J = 8.4 Hz, 1H), 8.34 (d, J = 8.4 Hz, 1H), 8.78 (bs, 1H); ^13^C NMR (DMSO-d_6_, 150 MHz) δ 25.1, 47.1, 115.2, 117.5, 122.2, 127.4, 128.6, 128.6, 135.4, 137.4, 137.8, 143.9, 144.1, 147.0, 148.0, 154.8, 164.7, 176.8, 181.6; HRAPCIMS *m*/*z* 420.1125 (calcd. for C_23_H_18_ClN_3_O_3_, 420.1114) (Figure S12).

6-chloro-2-methyl-7-{[2-(morpholin-4-yl)quinolin-8-yl]oxy}quinoline-5,8-dione (**12f**): 0.164 g (0.376 mmol); yield 91%; oil; ^1^H NMR (DMSO-d_6_, 600 MHz) δ 2.72 (s, 3H), 3.21 (m, 4H), 3.74 (m, 4H), 7.03 (d, J = 8.4, 1H), 7.29 (d, J = 8.4 Hz, 1H), 7.39 (d, J = 8.4 Hz, 1H), 7.79 (d, J = 8.4 Hz, 1H), 8.09 (d, J = 8.4 Hz, 1H), 8.11 (d, J = 8.4 Hz, 1H), 9.12 (bs, 1H); ^13^C NMR (DMSO-d_6_, 150 MHz) δ 25.1, 45.6, 110.9, 117.3, 123.5, 123.7, 126.4, 127.4, 128.6, 129.9, 135.4, 137.3, 138.0, 138.5, 144.1, 147.0, 148.6, 156.8, 164.7, 176.7, 181.6; HRAPCIMS m/z 436.1076 (calcd. for C_23_H_18_ClN_3_O_4_, 436.1064) (Figure S13).

6-chloro-7-(quinolin-8-yloxy)isoquinoline-5,8-dione (**13a**): 0.073 g (0.216 mmol); yield 49%; mp 86–88 °C; ^1^H NMR (DMSO-d_6_, 150 MHz) δ 7.59 (m, 1H), 7.88 (dd, J = 4.8 Hz, J = 8.4 Hz, 1H), 8.05 (d, J = 8.4 Hz, 1H), 8.33 (m, 2H), 9.10 (m, 2H), 9.47 (s, 1H); 9.49 (s, 1H); ^13^C NMR (DMSO-d_6_, 150 MHz) δ 117.8, 119.8, 122.0, 124.2, 126.1, 128.3, 128.5, 130.7, 133.6, 135.9, 147.3, 150.3, 154.9, 179.5; HRAPCIMS *m*/*z* 337.0379 (calcd. for C_18_H_9_ClN_2_O_3_, 337.0379) (Figure S14).

6-chloro-7-[(2-methylquinolin-8-yl)oxy]isoquinoline-5,8-dione (**13b**): 0.080 g (0.228 mmol); yield 52%, mp 110–112 °C; ^1^H NMR (CDCl_3_, 600 MHz) δ 2.97 (s, 3H), 7.57 (dd, J = 1.2 Hz, J = 8.4 Hz, 1H), 9.93 (d, J = 4.8 Hz, 1H), 8.17 (d, J = 8.4 Hz, 1H), 8.30 (dd, J = 7.8 Hz, J = 8.4 Hz, 1H), 8.39 (d, J = 8.4 Hz, 1H), 9.18 (d, J = 4.8 Hz, 1H), 9.55 (s, 1H), 9.58 (s, 1H); ^13^C NMR (CDCl_3_, 150 MHz) δ 24.4, 118.7, 119.0, 122.9, 123.7, 124.8, 125.9, 126.6, 130.3, 132.4, 136.1, 137.5, 146.2, 154.6, 159.8, 176.2, 180.0; HRAPCIMS *m*/*z* 351.0553 (calcd. for C_19_H_11_ClN_2_O_3_, 351.0536) (Figure S15).

8-[(6-chloro-5,8-dioxo-5,8-dihydroisoquinolin-7-yl)oxy]quinoline-2-carbaldehyde (**13c**): 0.111 g (0.299 mmol); yield 68%; mp 166–168 °C; ^1^H NMR (CDCl_3_, 600 MHz) δ 8.04 (m, 2H), 8.17 (d, J = 8.4 Hz, 1H), 8.28 (d, J = 8.4 Hz, 1H), 8.50 (m, 2H), 9.21 (d, J = 4.8 Hz, 1H), 9.56 (s, 1H), 10.41 (s, 1H); ^13^C NMR (CDCl_3_, 150 MHz) δ 111.3, 119.0, 119.3, 123.3, 123.6, 125.0, 125.6, 127.1, 131.0, 136.7, 138.1, 148.7, 152.8, 153.1, 156.3, 173.5, 180.9, 193.0; HRAPCIMS m/z 365.0317 (calcd. for C_19_H_9_ClN_2_O_4_, 365.0329) (Figure S16).

6-chloro-7-[(2-chloroquinolin-8-yl)oxy]isoquinoline-5,8-dione (**13d**): 0.098 g (0.269 mmol); yield 61%; mp 127–129 °C; ^1^H NMR (CDCl_3_, 600 MHz) δ 7.64 (d, J = 9.0 Hz, 1H), 7.96 (d, J = 8.4 Hz, 1H), 8.17 (m, 2H), 8.34 (d, J = 8.4 Hz, 1H), 8.46 (d, J = 8.4 Hz, 1H), 9.19 (d, J = 4.8 Hz, 1H), 9.54 (s, 1H); ^13^C NMR (CDCl_3_, 150 MHz) δ 28.7, 118.5, 120.4, 122.4, 123.5, 124.0, 124.9, 125.8, 127.0, 135.2, 137.4, 138.3, 147.2, 150.5, 152.4, 154.5, 179.7; HRAPCIMS *m*/*z* 371.006 (calcd. for C_18_H_8_Cl_2_N_2_O_3_, 370.999) (Figure S17).

6-chloro-7-{[2-(pyrrolidin-1-yl)quinolin-8-yl]oxy}isoquinoline-5,8-dione (**13e**): 0.150 g (0.370 mmol); yield 84%; oil; ^1^H NMR (CDCl_3_, 600 MHz) δ 2.10 (bs, 4H), 3.65 (bs, 4H), 6.85 (d, J = 8.4 Hz, 1H), 7.03 (d, J = 7.8 Hz, 1H), 7.27 (d, J = 8.4 Hz, 1H), 7.93 (dd, J = 7.8 Hz, 1H), 7.98 (d, J = 5.4 Hz, 1H), 8.04 (d, J = 5.4 Hz, 1H), 9.14 (dd, J = 5.4 Hz, J = 8.4 Hz, 1H), 9.46 (s, 1H); ^13^C NMR (CDCl_3_, 150 MHz) δ 25.5, 45.1, 112.0, 118.9, 119.2, 122.5, 124.8, 127.1, 130.2, 136.5, 144.0, 144.8, 149.5, 155.4, 177.9, 181.0; HRAPCIMS *m*/*z* 406.0959 (calcd. for C_22_H_16_ClN_3_O_3_, 406.0958) (Figure S18).

6-chloro-7-{[2-(morpholin-4-yl)quinolin-8-yl]oxy}isoquinoline-5,8-dione (**13f**): 0.165 g (0.391 mmol); yield 89%; oil; ^1^H NMR (DMSO-d_6_, 600 MHz) δ 3.79 (m, 8H), 7.06 (d, J = 8.4 Hz, 1H), 7.34 (d, J = 4.8 Hz, 1H), 7.37 (d, J = 8.4 Hz, 1H), 8.06 (d, J = 8.4 Hz, 1H), 8.15 (d, J = 8.4 Hz, 1H), 8.36 (bs, 1H), 9.20 (d, J = 4.8 Hz, 1H), 9.30 (s, 1H); ^13^C NMR (DMSO-d_6_, 150 MHz) δ 27.9, 45.6, 110.9, 111.5, 115.7, 117.4, 119.3, 123.5, 123.7, 125.3, 137.2, 138.01, 143.4, 145.1, 148.5, 156.8, 178.0, 181.5; HRAPCIMS *m*/*z* 422.0922 (calcd. for C_22_H_16_ClN_3_O_4_, 422.0908) (Figure S19).

2-chloro-3[(quinolin-8-yl)oxy]naphthalene-1,4-dione (**14a**): 0.093 g (0.278 mmol); yield 63%; mp 115–117 °C; ^1^H NMR (DMSO-d_6_, 600 MHz) δ 7.80 (dd, J = 4.2 Hz, J = 7.8 Hz, 1H), 7.97 (m, 2H), 8.16 (d, J = 9.0 Hz, 1H), 8.22 (m, 2H), 8.34 (d, J = 8.4 Hz, 1H), 8.67 (dd, J = 1.8 Hz, J = 9.0 Hz, 1H), 9.13 (dd, J = 1.8 Hz, J = 3.6 Hz, 1H), 10.24 (bs, 1H); ^13^C NMR (DMSO-d_6_, 150 MHz) δ 120.7, 121.1, 122.6, 122.7, 123.6, 127.1, 127.6, 129.2, 131.1, 132.3, 132.8, 133.5, 134.9, 137.5, 145.0, 152.0, 154.2, 181.8, 182.7; HRAPCIMS *m*/*z* 336.0431 (calcd. for C_19_H_10_ClNO_3_, 336.0427) (Figure S20).

2-chloro-3-[(2-methylquinolin-8-yl)oxy]naphthalene-1,4-dione (**14b**): 0.072 g (0.207 mmol); yield 47%; mp 100–102 °C; ^1^H NMR (DMSO-d_6_, 600 MHz) δ 2.85 (s, 3H), 7.72 (d, J = 8.4 Hz, 1H), 8.01 (m, 2H), 8.13 (d, J = 8.4 Hz, 1H), 8.26 (m, 2H), 8.28 (m, 2H), 8.57 (d, J = 8.4 Hz, 1H); ^13^C NMR (DMSO-d_6_, 150 MHz) δ 25.4, 119.7, 122.7, 124.5, 124.2, 126.9, 127.0, 127.4, 127.5, 132.8, 133.5, 134.9, 136.0, 136.1, 137.5, 154.1, 160.9, 181.8; HRAPCIMS *m*/*z* 350.0595 (calcd. for C_20_H_12_ClNO_3_, 350.0584) (Figure S21).

8-[(3-chloro-1,4-dioxo-1,4-dihydronaphthalen-2-yl)oxy]quinoline-2-carbaldehyde (**14c**): 0.120 g (0.330 mmol); yield 75%; mp 131–133 °C; ^1^H NMR (CDCl_3_, 600 MHz) δ 7.87 (m, 2H), 8.02 (d, J = 8.4 Hz, 1H), 8.26 (d, J = 8.4 Hz, 1H), 8.33 (m, 2H), 8.37 (m, 1H), 8.55 (d, J = 7.8 Hz, 1H), 8.59 (d, J = 9.0 Hz, 1H), 10.42 (s, 1H); ^13^C NMR (CDCl_3_, 150 MHz) δ 118.9, 123.7, 124.7, 126.6, 127.0, 127.3, 130.9, 132.6, 133.3, 134.4, 136.8, 138.1, 152.9, 154.1, 174.6, 181.5, 193.2; HRAPCIMS *m*/*z* 364.0350 (calcd. for C_20_H_10_ClNO_4_, 364.0377) (Figure S22).

2-chloro-3-[(2-chloroquinolin-8-yl)oxy]naphthalene-1,4-dione (**14d**): 0.132 g (0.357 mmol); yield 81%; mp 101–103 °C; ^1^H NMR (CDCl_3_, 600 MHz) δ 7.67 (m, 2H), 7.89 (d, J = 8.4 Hz, 1H), 7.93 (m, 1H), 7.98 (m, 2H), 8.19 (d, J = 7.2 Hz, 1H), 8.22 (m, 1H), 8.57 (d, J = 8.4 Hz, 1H); ^13^C NMR (CDCl_3_, 150 MHz) δ 113.9, 117.1, 123.9, 127.1, 128.0, 128.5, 129.0, 131.0, 131.7, 137.9, 140.9, 149.6, 151.1, 155.3, 178.0, 178.8; HRAPCIMS *m*/*z* 370.0040 (calcd. for C_19_H_9_Cl_2_NO_3_, 370.0038) (Figure S23).

2-chloro-3-{[2-(pyrrolidin-1-yl)quinolin-8-yl]oxy}naphthalene-1,4-dione (**14e**): 0.148 g (0.366 mmol); yield 83%; oil; ^1^H NMR (CDCl_3_, 600 MHz) δ 3.63 (bs, 8H), 7.00 (m, 2H), 7.06 (d, J = 7.8 Hz, 1H), 7.28 (d, J = 7.8 Hz, 1H), 7.93 (d, J = 8.4 Hz, 1H), 7.99 (m, 2H), 8.07 (d, J = 8.4 Hz, 1H), 8.21 (m, 1H); ^13^C NMR (CDCl_3_, 150 MHz) δ 25.4, 47.1, 110.1, 117.5, 120.4, 122.3, 122.7, 127.1, 127.3, 131.5, 134.9, 135.3, 137.5, 143.3, 145.2, 147.9, 151.4, 154.8, 178.3, 181.7; HRAPCIMS *m*/*z* 405.1022 (calcd. for C_23_H_17_ClN_2_O_3_, 405.1006) (Figure S24).

2-chloro-3-{[2-(morpholin-4-yl)quinolin-8-yl]oxy}naphthalene-1,4-dione (**14f**): 0.172 g (0.409 mmol); yield 93%; oil; ^1^H NMR (DMSO-d_6_, 600 MHz) δ 3.76 (m, 4H), 3.80 (m, 4H), 7.22 (d, J = 8.4, 1H), 7.32 (d, J = 8.4 Hz, 1H), 7.37 (d, J = 9.0 Hz, 1H), 8.00 (m, 2H), 8.08 (m, 1H), 8.15 (d, J = 9.0 Hz, 1H), 8.22 (m, 2H); ^13^C NMR (DMSO-d_6_, 150 MHz) δ 27.9, 45.6, 110.8, 117.3, 123.6, 127.1, 127.3, 131.6, 132.1, 134.9, 135.3, 137.2, 138.1, 143.4, 145.2, 148.5, 156.8, 178.3, 181.7; HRAPCIMS *m*/*z* 421.0937 (calcd. for C_23_H_17_ClN_2_O_4_, 421.0955) (Figure S25).

### 3.2. Biological Activity

#### 3.2.1. Enzymatic Assay

Compounds **11**–**14** (10 µmol/L) were tested as NQO1 substrates using the NADPH recycling assay according to the literature method [23,45]. The recombinant NQO1 (DT-diaphorase, EC 1.6.5.5, human recombinant, Sigma-Aldrich) was used, and the oxidation of NADPH to NADP^+^ was measured at the absorption wavelength of 340 nm on the BioTek 800TS microplate reader (BioKom, Janki, Poland). Compounds were dissolved in dimethyl sulfoxide (2 µL) and added to the 96-well plate. The NQO1 protein (1.4 µg/mL) in 50 mmol/L potassium phosphate buffer (pH = 7.4) was added to each well (198 µL) (Nunc Thermo Fisher Scientific, Waltham, MA, USA). Once the 96-well plate was filled with the assay solutions, except the NADPH solution, it was placed into the instrument and left to sit for 5 min before starting the measurements. The enzymatic reaction was initiated by automatically dispensing the NADPH solution into the wells, and data were recorded at 10 s intervals for 5 min at 25 °C. The linear part of the absorbance vs. time graphs (the first 20 s to 1 min) was fitted, and the slopes were calculated. NADPH oxidation rates were compared with reactions lacking the tested compound. Initial velocities were calculated, and data were expressed as µmol NADPH/µmol NQO1/min. All reactions were carried out at least in triplicate.

#### 3.2.2. Anticancer Activity

Compounds **7**–**14** and doxorubicin were tested for cytotoxic activity in vitro against panel of human cancer cell lines: melanoma (Colo-829, ATCC, Rockville, MD, USA), ovarian cancer (SK-OV-3, ATCC, Rockville, MD, USA), breast cancer (T47D, MCF-7, and MDA-MB-231, ATCC, Rockville, MD, USA) and lung cancer (A549, ATCC, Rockville, MD, USA). The cultured cells were kept at 37 °C and 5% CO_2_. The cells were seeded (5 × 10^4^ cells/well/100 mL DMEM supplemented with 10% FCS and streptomycin/penicillin) using 96-well plates (Nunc Thermo Fisher Scientific, Waltham, MA, USA). The tested compounds **7**–**14** with the concentration of 1–100 µg/mL DMSO were inducted with the cancer cells for 72 h. The WST-1-formazan (proliferation reagent WST-1 assay, Roche Diagnostics, Mannheim, Germany) was detected using a microplate reader at 450 nm. Results were expressed as a mean value of at least three independent experiments performed in triplicate.

#### 3.2.3. Apoptotic Assay

Transcriptional activity of genes (TP53, CDKN1A, BAX, and BCL-2) was rated by real-time RT-QPCR using Opticon™ DNA Engine system (MJ Research, Watertown, NY, USA) and QuantTect^®^ SYBR^®^ Green RT-PCR Kit (Qiagen, Hilden, Germany). Cultured cells were incubated with tested compounds for 24 h. RNA was extracted using Quick-RNA™ MiniPrep kit columns (Zymo Research, Irvine, CA, USA). The extracted RNA was assessed qualitatively and quantitatively. The amount and purity of the total RNA in extracts were determined spectrophotometrically (HP8452A apparatus, Hewlett Packard, Waldbronn, Germany).

### 3.3. Molecular Docking

A molecular docking study was performed using the crystal structure of human NQO1 protein, which was collected from the Protein Data Bank (PDB) database with the PDB identifier 2F1O [46]. During docking, the FAD molecules were presented into binding sites as cofactors.

A molecular docking study was performed with the AutoDock Vina software package [47]. The grid center of Vina docking was selected as the center of reference ligands that accompanied the downloaded protein complexes. The grid size was set to 25 Å × 25 Å × 25 Å, which is large enough to cover the entire target active site. Default values of all other parameters were used, and the complexes were submitted to 8 genetic algorithm runs. All obtained results were visualized using the BIOVIA Discovery Studio software package [48].

### 3.4. Statistical Analysis

Mann–Whitney *U* test was used to determine statistical differences between the means, where *p* < 0.05 was considered to be statistically significant.

## 4. Conclusions

The combination of 1,4-quinone with a quinoline moiety allowed us to obtain a new class of compounds as potential anticancer agents. The enzymatic assay showed that the new hybrids are suitable substrates for the NQO1 protein. It was found that the enzymatic conversion rate depends on the type of 1,4-quinone moiety. Most of the hybrids **11**–**14** exhibited lower cytotoxicity in vitro than doxorubicin against normal cell lines (HFF-1). The structure-activity relationship showed that the nitrogen atom is necessary to preserve the antiproliferative effect. It should be noted that hybrids showing the highest activity against the lung cancer cell line (A549) also have an overexpression of the gene encoding the NQO1 protein. The selected derivatives were tested for their influence on the expression of H3, TP53, CDKN1A, BAX, and BCL-2 genes. It was shown that the tested compounds are able to activate the mitochondrial apoptosis pathway in A549 cells. A molecular docking study was used to examine the interaction between NQO1 protein and selected hybrids. The results showed that the 1,4-quinone moiety interacts with the enzyme active site through hydrogen bonding and hydrophobic interactions.

## Figures and Tables

**Table 1:** The biological activity of compounds **11**–**14** and doxorubicin.

Compound	Cell Line/IC_50_ (µM)						
	Colo-829	SK-OV-3	MDA-MB-231	T47D	MCF-7	A549	HFF-1
**11a**	18.51 ± 1.31	1.55 ± 0.20	11.49 ± 0.19	18.68 ± 0.71	19.35 ± 0.37	1.68 ± 0.05	10.21 ± 0.16
**11b**	1.92 ± 0.11	1.69 ± 0.03	1.64 ± 0.15	2.42 ± 0.13	2.11 ± 0.08	1.78 ± 0.21	26.06 ± 1.02
**11c**	11.65 ± 1.36	1.78 ± 0.31	14.35 ± 2.01	22.19 ± 0.66	18.33 ± 1.23	1.45 ± 0.13	10.52 ± 0.87
**11d**	1.45 ± 0.24	1.56 ± 0.11	2.03 ± 0.05	2.10 ± 0.11	2.11 ± 0.09	1.71 ± 0.03	13.74 ± 1.27
**11e**	15.01 ± 0.32	1.72 ± 0.09	1.97 ± 0.13	20.79 ± 1.03	2.03 ± 0.03	12.12 ± 1.06	11.45 ± 0.69
**11f**	14.04 ± 1.16	10.89 ± 0.48	13.75 ± 1.09	18.62 ± 0.62	16.21 ± 0.12	12.63 ± 0.82	25.64 ± 0.19
**12a**	1.52 ± 0.35	0.05 ± 0.02	1.97 ± 0.15	1.59 ± 0.15	2.09 ± 0.02	0.85 ± 0.04	17.34 ± 0.89
**12b**	1.69 ± 0.08	1.49 ± 0.21	1.57 ± 0.19	1.90 ± 0.23	1.78 ± 0.03	1.43 ± 0.06	10.15 ± 1.33
**12c**	14.55 ± 0.51	2.16 ± 0.19	21.12 ± 0.11	23.26 ± 1.12	19.11 ± 0.95	3.14 ± 0.43	17.89 ± 1.37
**12d**	13.12 ± 0.49	15.38 ± 0.48	15.55 ± 0.79	16.17 ± 0.98	17.41 ± 0.82	1.46 ± 0.06	10.28 ± 1.65
**12e**	1.53 ± 0.08	1.05 ± 0.06	1.50 ± 0.15	17.35 ± 1.30	1.89 ± 0.23	15.12 ± 0.39	17.56 ± 1.05
**12f**	13.35 ± 0.21	13.49 ± 0.21	10.22 ± 0.28	17.34 ± 0.26	14.11 ± 0.67	11.56 ± 0.72	19.93 ± 0.93
**13a**	0.17 ± 0.02	0.46 ± 0.02	1.02 ± 0.15	0.09 ± 0.04	1.16 ± 0.15	0.03 ± 0.01	27.69 ± 0.70
**13b**	0.39 ± 0.11	0.49 ± 0.05	0.11 ± 0.01	1.29 ± 0.03	0.61 ± 0.09	0.57 ± 0.02	26.04 ± 1.60
**13c**	0.34 ± 0.28	0.32 ± 0.01	0.75 ± 0.09	0.52 ± 0.08	0.89 ± 0.03	1.32 ± 0.32	13.25 ± 0.85
**13d**	1.96 ± 0.08	17.08 ± 1.76	1.67 ± 0.11	Neg.	1.75 ± 0.19	0.06 ± 0.01	11.09 ± 1.07
**13e**	19.57 ± 1.32	15.85 ± 0.26	16.61 ± 0.38	Neg.	16.85 ± 0.50	14.04 ± 0.26	15.52 ± 0.72
**13f**	2.10 ± 0.06	1.23 ± 0.08	0.13 ± 0.05	2.15 ± 0.06	1.03 ± 0.19	1.22 ± 0.08	18.39 ± 0.35
**14a**	15.30 ± 2.56	1.70 ± 0.29	15.22 ± 0.85	Neg.	14.07 ± 0.98	0.65 ± 0.07	12.81 ± 0.85
**14b**	15.04 ± 1.03	16.78 ± 0.59	19.15 ± 0.62	22.16 ± 1.03	16.31 ± 1.08	2.10 ± 0.29	11.14 ± 1.62
**14c**	Neg.	0.25 ± 0.06	25.14 ± 0.97	1.62 ± 0.21	Neg.	0.41 ± 0.06	17.96 ± 1.75
**14d**	17.98 ± 2.01	14.12 ± 0.52	2.05 ± 0.35	Neg.	11.66 ± 1.34	8.13 ± 1.02	14.32 ± 1.23
**14e**	22.00 ± 0.60	15.92 ± 0.22	7.66 ± 1.12	Neg.	24.05 ± 0.21	22.14 ± 0.89	18.65 ± 1.36
**14f**	11.35 ± 1.97	14.16 ± 0.76	2.35 ± 0.09	Neg.	17.45 ± 1.02	14.02 ± 0.86	20.30 ± 0.87
**Doxorubicin**	0.05 ± 0.01	0.15 ± 0.03	0.64 ± 0.06	0.04 ± 0.01	0.12 ± 0.05	0.02 ± 0.01	0.12 ± 0.03

Neg. means that IC_50_ > 30 µM.

**Table 2:** Scoring values (ΔG) of hybrids **1**–**24** and streptonigrin (**ST**).

Ligand	ΔG (kcal/mol)	Ligand	ΔG (kcal/mol)
**11a**	−10.1	**13a**	−10.4
**11b**	−10.4	**13b**	−10.7
**11c**	−10.3	**13c**	−9.9
**11d**	−10.0	**13d**	−10.9
**11e**	−10.9	**13e**	−10.6
**11f**	−10.1	**13f**	−10.8
**12a**	−10.3	**14a**	−10.3
**12b**	−10.6	**14b**	−10.1
**12c**	−10.5	**14c**	−9.7
**12d**	−10.6	**14d**	−9.5
**12e**	−10.2	**14e**	−9.4
**12f**	−10.3	**14f**	−9.6
**ST**	−7.1		

## Data Availability

Not applicable.

## Supplementary Materials

The following supporting information can be downloaded at: https://www.mdpi.com/article/10.3390/molecules27196206/s1, Table S1: The NQO1 activity of hybrids **11**–**14** and ST monitored at the absorbance of A340 nm.; Table S2: The selectivity index (SI) value for compounds **11**–1**4** and cisplatin.; Table S3: Interaction of selected hybrids with the active site of NQO1 protein.; Figure S1. Visualization of hydrogen bond (green) and hydrophobic interactions (violet) between NQO1 enzyme and hybrid: (**a**) **11b**; (**b**) **12b**; (**c**) **13b**; (**d**) **14b**; Figure S12: Spectra of 6-chloro-7-(quinoline-8-yloxy)quinoline-5,8-dione (**11a**).; Figure S3: Spectra of 6-chloro-7-[(2-methylquinolin-8-yl)oxy]quinoline-5,8-dione (**11b**).; Figure S4: Spectra of 8-[(6-chloro-5,8-dioxo-5,8-dihydroquinolin-7-yl)oxy]quinoline-2-carbaldehyde (**11c**).; Figure S5: Spectra of 6-chloro-7-[(2-chloroquinolin-8-yl)oxy]quinoline-5,8-dione (**11d**).; Figure S6: Spectrum of 6-chloro-7-{[2-(pyrrolidin-1-yl)quinolin-8-yl]oxy}quinoline-5,8-dione (**11e**).; Figure S7: Spectra of 6-chloro-7-{[2-(morpholin-4-yl)quinolin-8-yl]oxy}quinoline-5,8-dione (**11f**).; Figure S8: Spectra of 6-chloro-2-methyl-7-(quinoline-8-yloxy)quinoline-5,8-dione (**12a**).; Figure S9: Spectra of 6-chloro-2-methyl-7-[(2-methylquinolin-8-yl)oxy]quinoline-5,8-dione (**12b**).; Figure S10: Spectra of 8-[(6-chloro-2-methyl-5,8-dioxo-5,8-dihydroquinolin-7-yl)oxy]quinoline-2-carbaldehyde (**12c**).; Figure S11: Spectra of 6-chloro-2-methyl-7-[(2-chloroquinolin-8-yl)oxy]quinoline-5,8-dione (**12d**).; Figure S12: Spectra of 6-chloro-2-methyl-7-{[2-(pyrrolidin-1-yl)quinolin-8-yl]oxy}quinoline-5,8-dione (**12e**).; Figure S13: Spectra of 6-chloro-2-methyl-7-{[2-(morpholin-4-yl)quinolin-8-yl]oxy}quinoline-5,8-dione (**12f**).; Figure S14: Spectra of 6-chloro-7-(quinoline-8-yloxy)isoquinoline-5,8-dione (**13a**).; Figure S15: Spectra of 6-chloro-7-[(2-methylquinolin-8-yl)oxy]isoquinoline-5,8-dione (**13b**).; Figure S16: Spectra of 8-[(6-chloro-5,8-dioxo-5,8-dihydroquinolin-7-yl)oxy]isoquinoline-2-carbaldehyde (**13c**).; Figure S17: Spectra of 6-chloro-7-[(2-chloroquinolin-8-yl)oxy]isoquinoline-5,8-dione (**13d**).; Figure S18: Spectra of 6-chloro-7-{[2-(pyrrolidin-1-yl)quinolin-8-yl]oxy}isoquinoline-5,8-dione (**13e**).; Figure S19: Spectra of 6-chloro-7-{[2-(morpholin-4-yl)quinolin-8-yl]oxy}isoquinoline-5,8-dione (**13f**).; Figure S20: Spectra of 2-chloro-3-[(quinolin-8-yl)oxy]naphthalene-1,4-dione (**14a**).; Figure S21: Spectra of 2-chloro-3-[(2-methylquinolin-8-yl)oxy]naphthalene-1,4-dione (**14b**).; Figure S22: Spectra of 8-[(3-chloro-1,4-dioxo-1,4-dihydronaphthalen-2-yl)oxy]quinoline-2-carbaldehyde (**14c**).; Figure S23: Spectra of 2-chloro-3-[(2-chloroquinolin-8-yl)oxy]naphthalene-1,4-dione (**14d**).; Figure S24: Spectra of 2-chloro-3-{[2-(pyrrolidin-1-yl)quinolin-8-yl]oxy}naphthalene-1,4-dione (**14e**).; Figure S25: Spectra of 2-chloro-3-{[2-(morpholin-4-yl)quinolin-8-yl]oxy}naphthalene-1,4-dione (**14f**).

## Sample Availability

Samples of compounds **1**–**14** are available from the authors.
