# Supplementary material for: Hybrids of 1,4-Quinone with Quinoline Derivatives: Synthesis, Biological Activity, and Molecular Docking with DT-Diaphorase (NQO1)
Source: Molecules. 2022 Sep 21;27(19):6206. doi: 10.3390/molecules27196206 (PMC9572083; doi:10.3390/molecules27196206)

Supplementary Materials

Hybrids of 1,4-quinone with quinoline derivatives: synthesis, biological activity and molecular docking with DT-diaphorase

Monika Kadela-Tomanek, Maria Jastrzębska, Elwira Chrobak, Ewa Bębenek, Małgorzata Latocha

Table of contects

Table S1: The NQO1 activity of hybrids **11-14** and **ST** monitored at the absorbance of A340 nm. 2
Table S2: The selectivity index (SI) value for compounds **11-14** and doxorubicin. 3
Table S3: Interaction of selected hybrids with active site of NQO1 protein. 4
Figure S1. Visualization of hydrogen bond (green) and hydrophobic interactions (violet) between NQO1 enzyme and hybrid: (**a**) **11b**; (**b**) **12b**; (**c**) **13b**; (**d**) **14b**. 5
Figure S2: Spectra of 6-chloro-7-(quinolin-8-yloxy)quinoline-5,8-dione (**11a**). 6
Figure S3: Spectra of 6-chloro-7-[(2-methylquinolin-8-yl)oxy]quinoline-5,8-dione (**11b**) 7
Figure S4: Spectra of 8-[(6-chloro-5,8-dioxo-5,8-dihydroquinolin-7-yl)oxy]quinoline-2-carbaldehyde (**11c**). 8
Figure S5: Spectra of 6-chloro-7-[(2-chloroquinolin-8-yl)oxy]quinoline-5,8-dione (**11d**). 9
Figure S6: Spectrum of 6-chloro-7-{[2-(pyrrolidin-1-yl)quinolin-8-yl]oxy}quinoline-5,8-dione (**11e**). ... 10
Figure S7: Spectra of 6-chloro-7-{[2-(morpholin-4-yl)quinolin-8-yl]oxy}quinoline-5,8-dione (**11f**). 11
Figure S8: Spectra of 6-chloro-2-methyl-7-(quinolin-8-yloxy)quinoline-5,8-dione (**12a**). 12
Figure S9: Spectra of 6-chloro-2-methyl-7-[(2-methylquinolin-8-yl)oxy]quinoline-5,8-dione (**12b**) 13
Figure S10: Spectra of 8-[(6-chloro-2-methyl-5,8-dioxo-5,8-dihydroquinolin-7-yl)oxy]quinoline-2-carbaldehyde (**12c**). 14
Figure S11: Spectra of 6-chloro-2-methyl-7-[(2-chloroquinolin-8-yl)oxy]quinoline-5,8-dione (**12d**). 15
Figure S12: Spectra of 6-chloro-2-methyl-7-{[2-(pyrrolidin-1-yl)quinolin-8-yl]oxy}quinoline-5,8-dione (**12e**). 16
Figure S13: Spectra of 6-chloro-2-methyl-7-{[2-(morpholin-4-yl)quinolin-8-yl]oxy}quinoline-5,8-dione (**12f**). 17
Figure S14: Spectra of 6-chloro-7-(quinolin-8-yloxy)isoquinoline-5,8-dione (**13a**). 18
Figure S15: Spectra of 6-chloro-7-[(2-methylquinolin-8-yl)oxy]isoquinoline-5,8-dione (**13b**) 19
Figure S16: Spectra of 8-[(6-chloro-5,8-dioxo-5,8-dihydroquinolin-7-yl)oxy]isoquinoline-2-carbaldehyde (**13c**). 20
Figure S17: Spectra of 6-chloro-7-[(2-chloroquinolin-8-yl)oxy]isoquinoline-5,8-dione (**13d**). 21
Figure S18: Spectra of 6-chloro-7-{[2-(pyrrolidin-1-yl)quinolin-8-yl]oxy}isoquinoline-5,8-dione (**13e**). 22
Figure S19: Spectra of 6-chloro-7-{[2-(morpholin-4-yl)quinolin-8-yl]oxy}isoquinoline-5,8-dione (**13f**). 23
Figure S20: Spectra of 2-chloro-3-[(quinolin-8-yl)oxy]naphthalene-1,4-dione (**14a**). 24
Figure S21: Spectra of 2-chloro-3-[(2-methylquinolin-8-yl)oxy]naphthalene-1,4-dione (**14b**). 25
Figure S22: Spectra of 8-[(3-chloro-1,4-dioxo-1,4-dihydronaphthalen-2-yl)oxy]quinoline-2-carbaldehyde (**14c**). 26
Figure S23: Spectra of 2-chloro-3-[(2-chloroquinolin-8-yl)oxy]naphthalene-1,4-dione (**14d**). 27
Figure S24: Spectra of 2-chloro-3-{[2-(pyrrolidin-1-yl)quinolin-8-yl]oxy}naphthalene-1,4-dione (**14e**). 28
Figure S25: Spectra of 2-chloro-3-{[2-(morpholin-4-yl)quinolin-8-yl]oxy}naphthalene-1,4-dione (**14f**). 29

Table S1: The NQO1 activity of hybrids **11-14** and **ST** monitored at the absorbance of A340 nm.

**Compound**	**NQO1 activity [µmol NADPH/µmol NQO1/min]**
**11a**	1399 ± 26
**12a**	1505 ± 54
**13a**	1210 ± 23
**14a**	1282 ± 29
**11b**	1294 ± 51
**12b**	1437 ± 42
**13b**	1143 ± 36
**14b**	1260 ± 15
**11c**	1100 ± 35
**12c**	470 ± 22
**13c**	318 ± 19
**14c**	550 ± 62
**11d**	800 ± 64
**12d**	890 ± 21
**13d**	633 ± 32
**14d**	1001 ± 43
**11e**	1082 ± 51
**12e**	600 ± 35
**13e**	214 ± 21
**14e**	354 ± 22
**11f**	113 ± 19
**12f**	210 ± 20
**13f**	143 ± 26
**14f**	87 ± 15
**ST**	725 ± 84

Table S2: The selectivity index (SI) value for compounds **11-14** and doxorubicin.

**Compound**	**SI ($IC_{50\ HFF-1}/IC_{50\ cancer\ line}$)**					
	**Colo-829**	**SK-OV-3**	**MDA-MB-231**	**T47D**	**MCF-7**	**A549**
**11a**	0.55	6.59	0.89	0.55	0.53	6.08
**11b**	13.56	15.40	15.87	10.76	12.34	14.62
**11c**	0.90	5.91	0.73	0.47	0.57	7.26
**11d**	9.48	8.81	6.77	6.54	6.51	8.04
**11e**	0.76	6.66	5.81	0.55	5.64	0.94
**11f**	1.83	2.35	1.86	1.38	1.58	2.03
**12a**	11.41	346.80	8.80	10.91	8.30	20.40
**12b**	6.01	6.81	6.46	5.34	5.70	7.10
**12c**	1.23	8.28	0.85	0.77	0.94	5.70
**12d**	0.78	0.67	0.66	0.64	0.59	7.04
**12e**	11.48	16.72	11.71	1.01	9.29	1.16
**12f**	1.49	1.48	1.95	1.15	1.41	1.72
**13a**	162.88	60.20	27.15	307.67	23.87	923.00
**13b**	66.77	53.14	236.73	20.19	42.69	45.68
**13c**	38.97	41.41	17.67	25.48	14.89	10.04
**13d**	5.66	0.65	6.64	-	6.34	184.83
**13e**	0.79	0.98	0.93	-	0.92	1.11
**13f**	8.76	14.95	141.46	8.55	17.85	15.07
**14a**	0.84	7.54	0.84	-	0.91	19.71
**14b**	0.74	0.66	0.58	0.50	0.68	5.30
**14c**	-	71.84	0.71	11.09	-	43.80
**14d**	0.80	1.01	6.99	-	1.23	1.76
**14e**	0.85	1.17	2.43	-	0.78	0.84
**14f**	1.98	1.59	9.58	-	1.29	1.61
**Doxorubicin**	2.40	0.80	0.19	3.00	1.00	6.00

Table S3: Interaction of selected hybrids with active site of NQO1 protein.

Ligand	H-bonding residues and length (Å)	π-interaction residues and length (Å)
**11b**	TYR128 (2.143)<br>GLY149 (2.902; 2.870)	TYR128 (3.746; 4.729; 5.497)<br>PHE178 (4.802; 5.482)<br>TRP105 (5.420; 4.795)<br>MET154 (4.574)<br>HIS161 (3.937)<br>FAD (4.050; 3.530; 3.934; 4.444; 5.139; 5.051)
**12b**	TYR128 (2.095)	TYR128 (3.726; 4.686; 5.419)<br>TYR126 (4.296)<br>PHE178 (5.532; 4.918)<br>TRP105 (3.873; 3.832)<br>MET154 (4.410)<br>HIS161 (3.860)<br>FAD (3.856; 3.603; 3.942;4.555; 3.840; 5.081; 5.278; 4.202)
**13b**	TYR128 (2.117)<br>GLY149 (2.918; 2.857)	TYR128 (3.744; 4.717; 5.505)<br>PHE178 (5.473; 4.800)<br>TRP105 (5.423; 4.796)<br>MET154 (4.580)<br>HIS161 (3.898)<br>FAD (3.528; 4.060; 4.443; 3.940; 5.141; 5.026)
**14b**	TYR128 (2.061)	TYR128 (4.344; 3.572; 5.104; 4.488)<br>TYR126 (4.675)<br>PHE178 (5.807; 5.126)<br>PHE236 (4.688)<br>TRP105 (5.800; 5.245)<br>MET154 (4.468; 5.251)<br>HIS161 (4.340)<br>FAD (3.780; 4.016; 4.602; 3.818; 4.992)

Figure S1. Visualization of hydrogen bond (green) and hydrophobic interactions (violet) between NQO1 enzyme and hybrid: (**a**) **11b**; (**b**) **12b**; (**c**) **13b**; (**d**) **14b**.

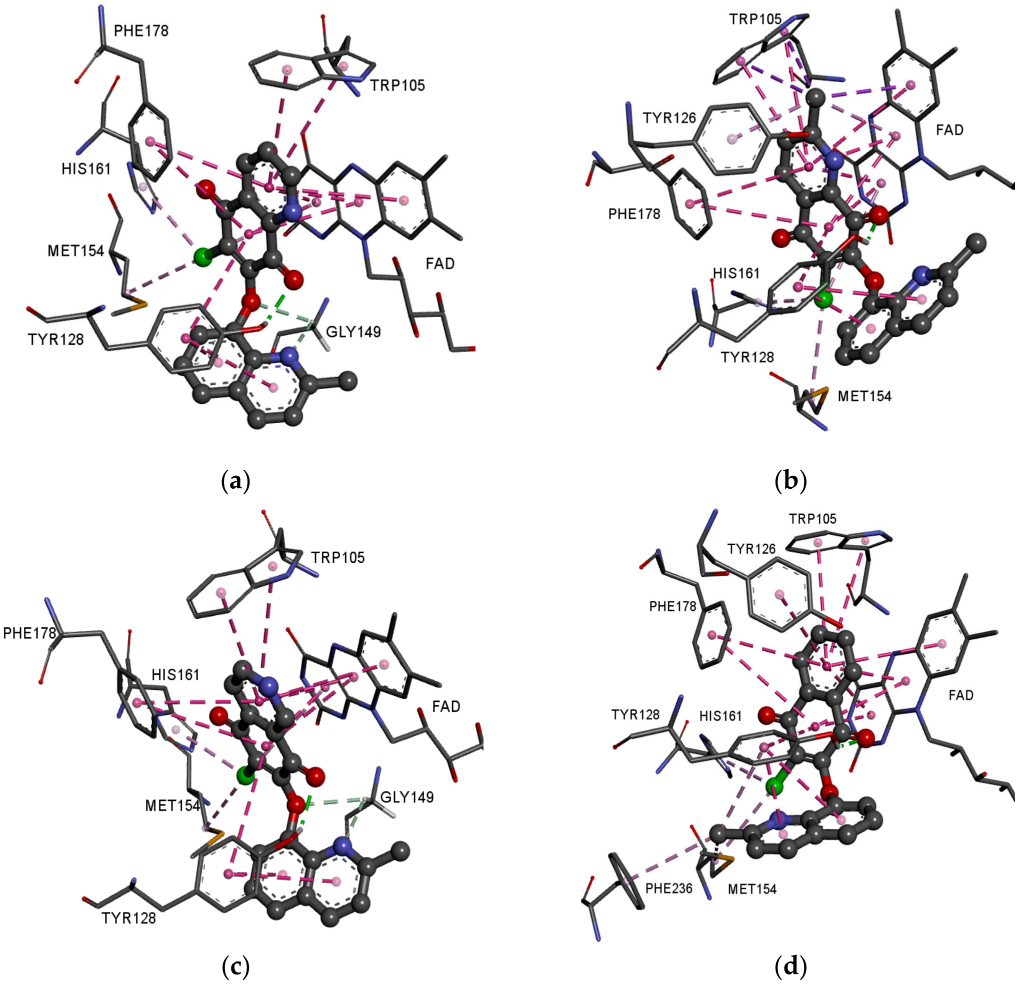

Figure S2: Spectra of 6-chloro-7-(quinolin-8-yloxy)quinoline-5,8-dione (**11a**).

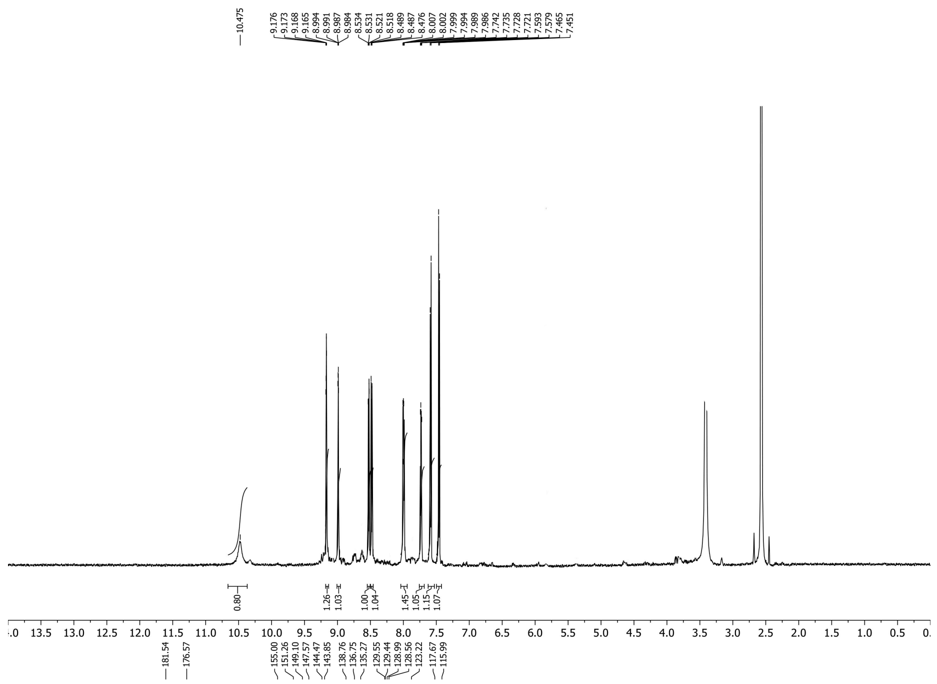

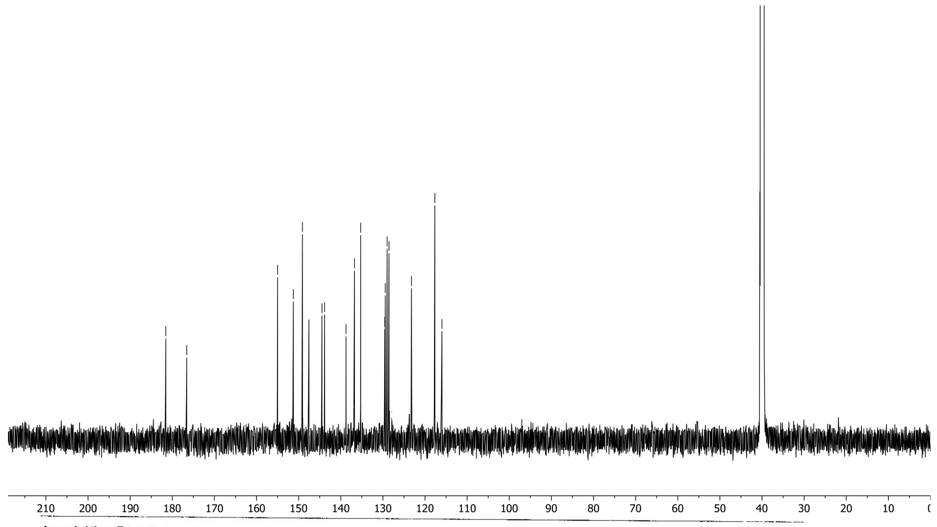

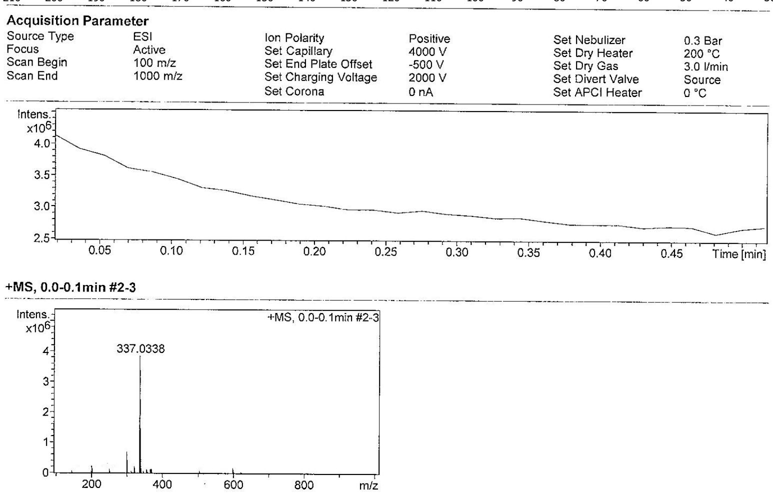

Figure S3: Spectra of 6-chloro-7-[(2-methylquinolin-8-yl)oxy]quinoline-5,8-dione (**11b**)

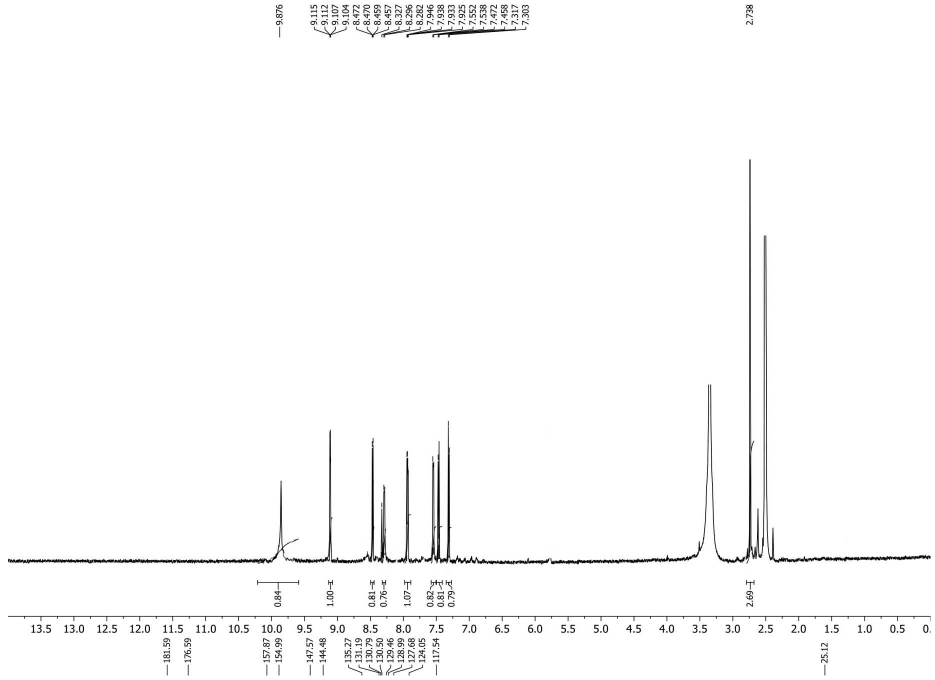

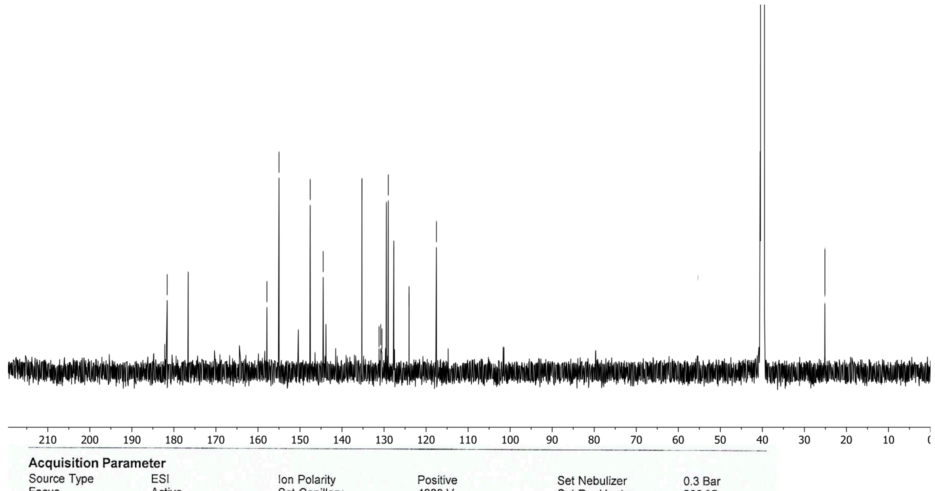

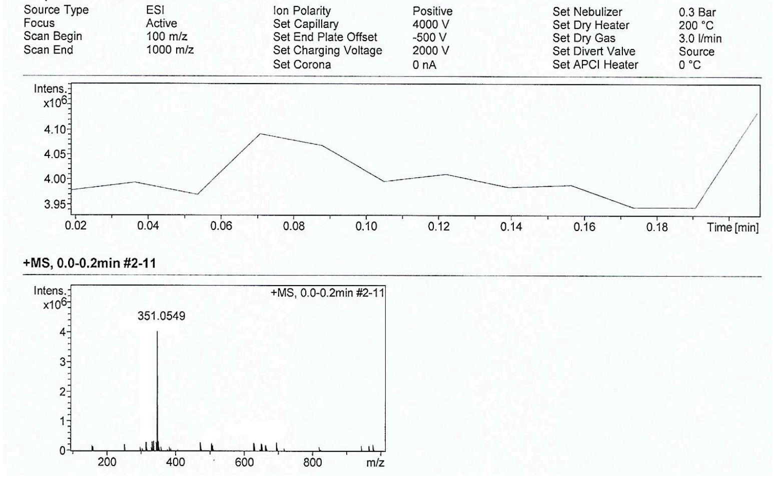

Figure S4: Spectra of 8-[(6-chloro-5,8-dioxo-5,8-dihydroquinolin-7-yl)oxy]quinoline-2-carbaldehyde (**11c**).

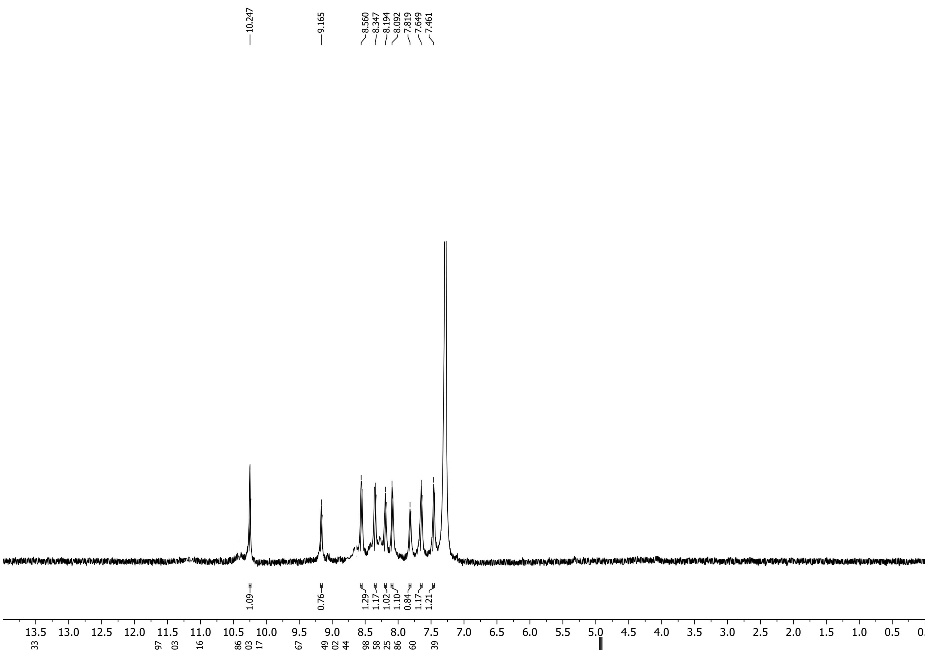

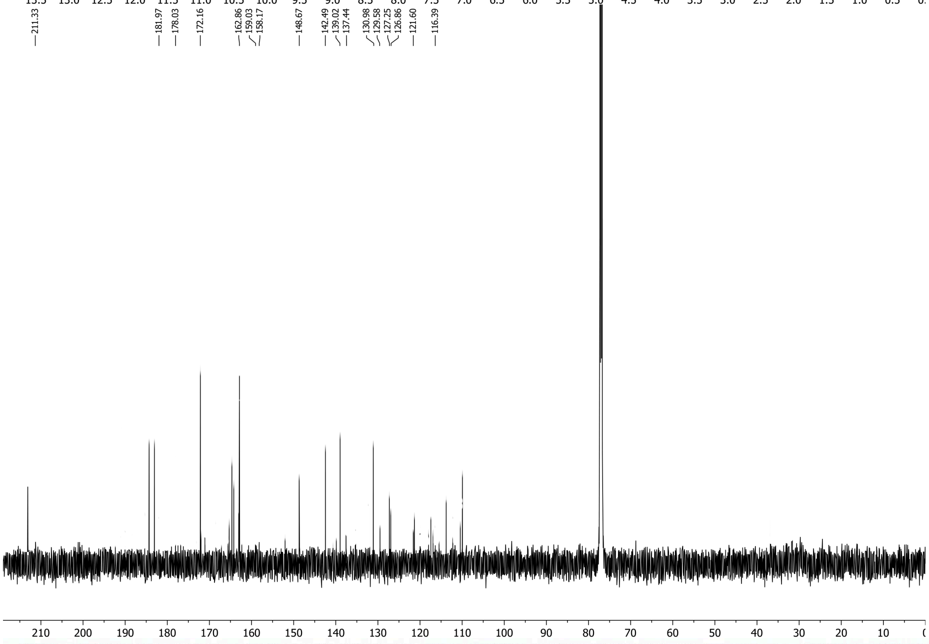

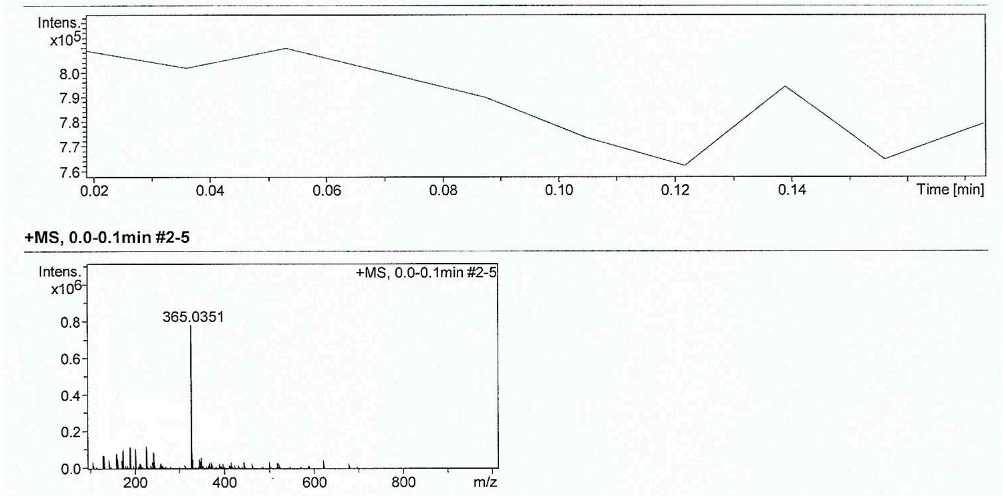

Figure S5: Spectra of 6-chloro-7-[(2-chloroquinolin-8-yl)oxy]quinoline-5,8-dione (**11d**).

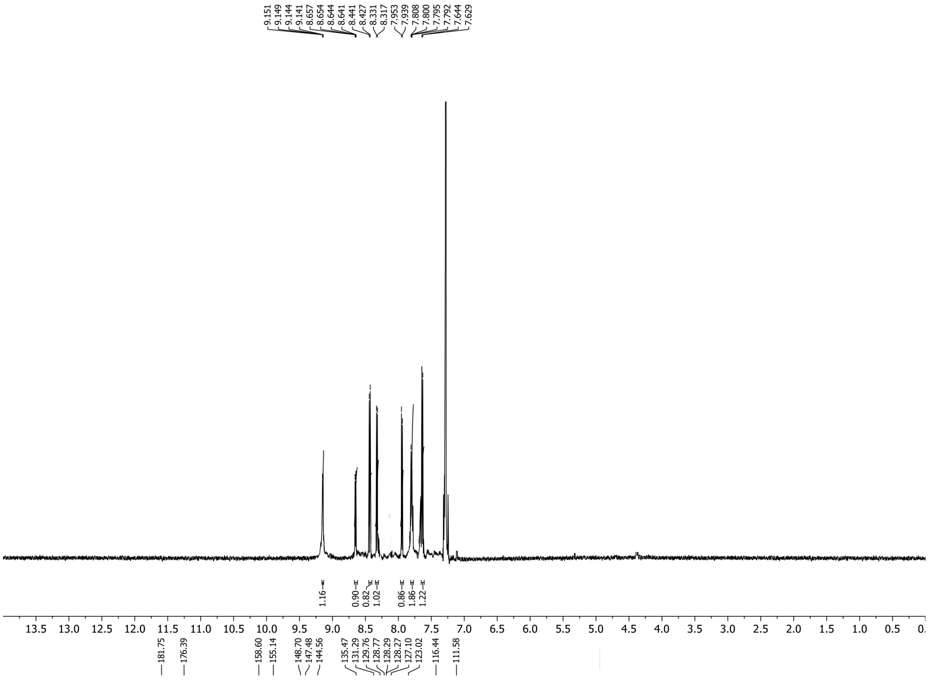

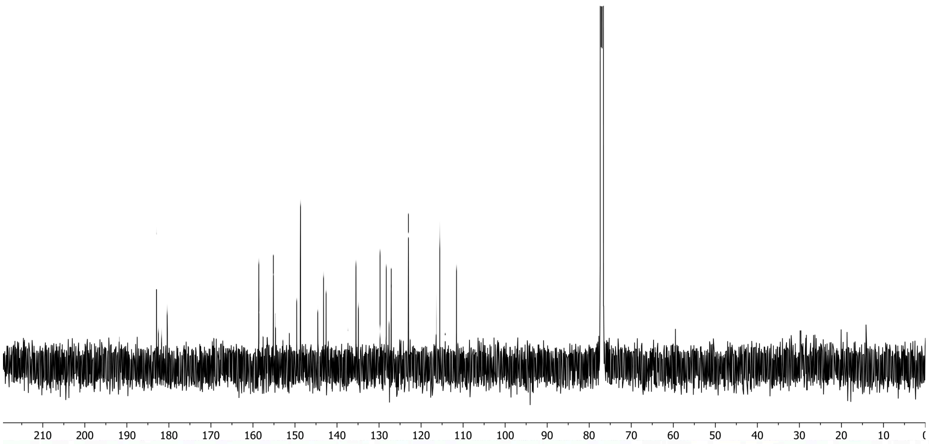

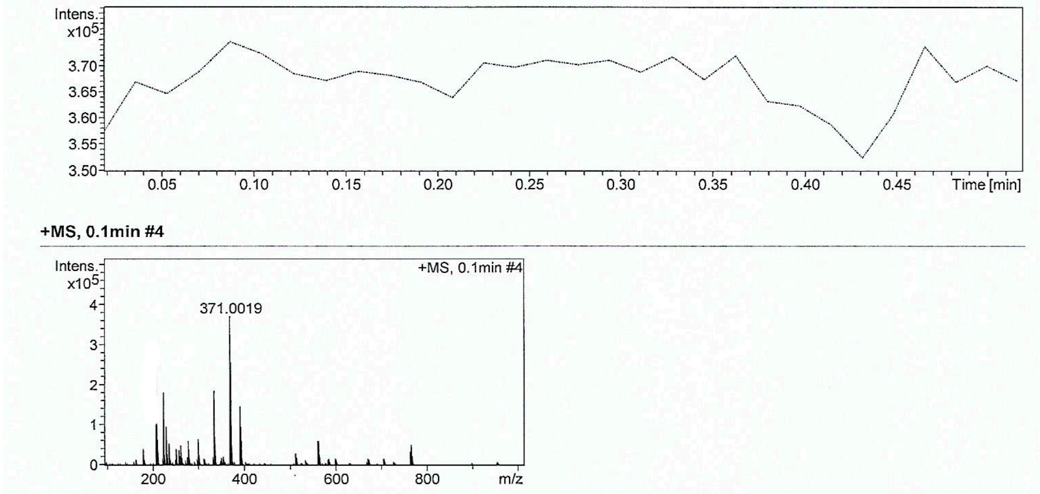

Figure S6: Spectrum of 6-chloro-7-{[2-(pyrrolidin-1-yl)quinolin-8-yl]oxy}quinoline-5,8-dione (**11e**).

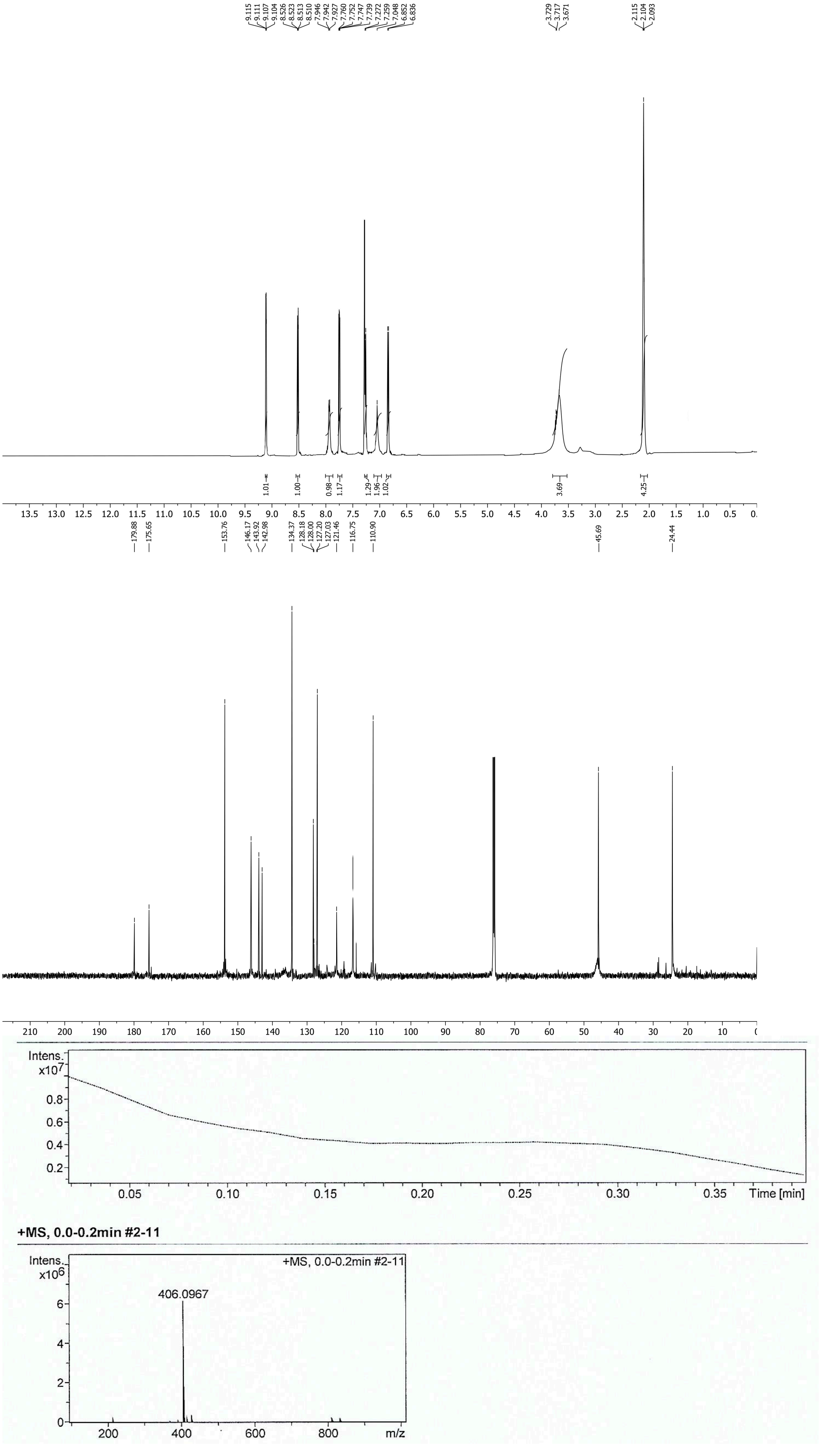

Figure S7: Spectra of 6-chloro-7-{[2-(morpholin-4-yl)quinolin-8-yl]oxy}quinoline-5,8-dione (**11f**).

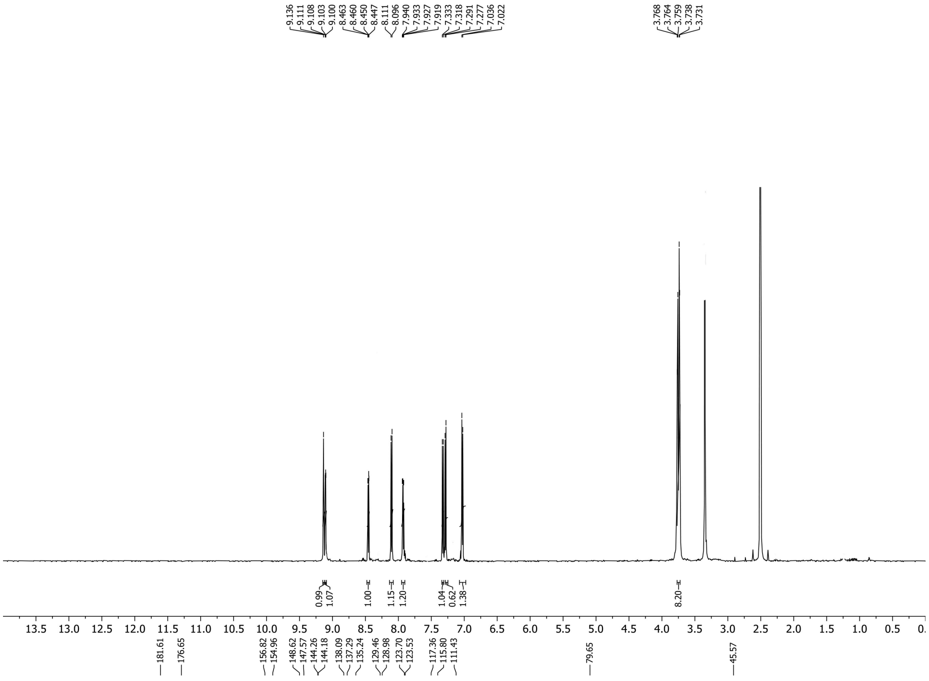

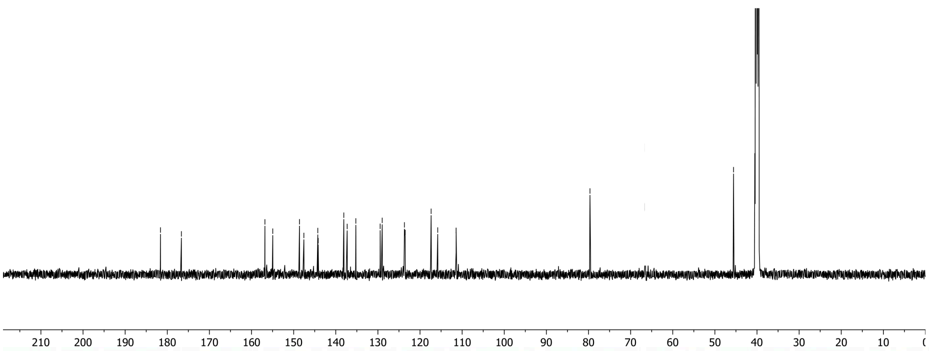

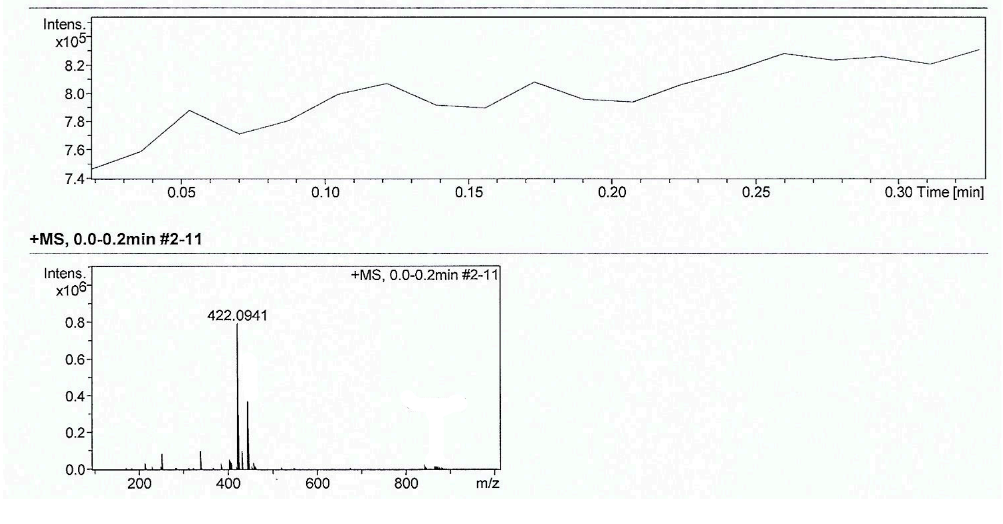

Figure S8: Spectra of 6-chloro-2-methyl-7-(quinolin-8-yloxy)quinoline-5,8-dione (**12a**).

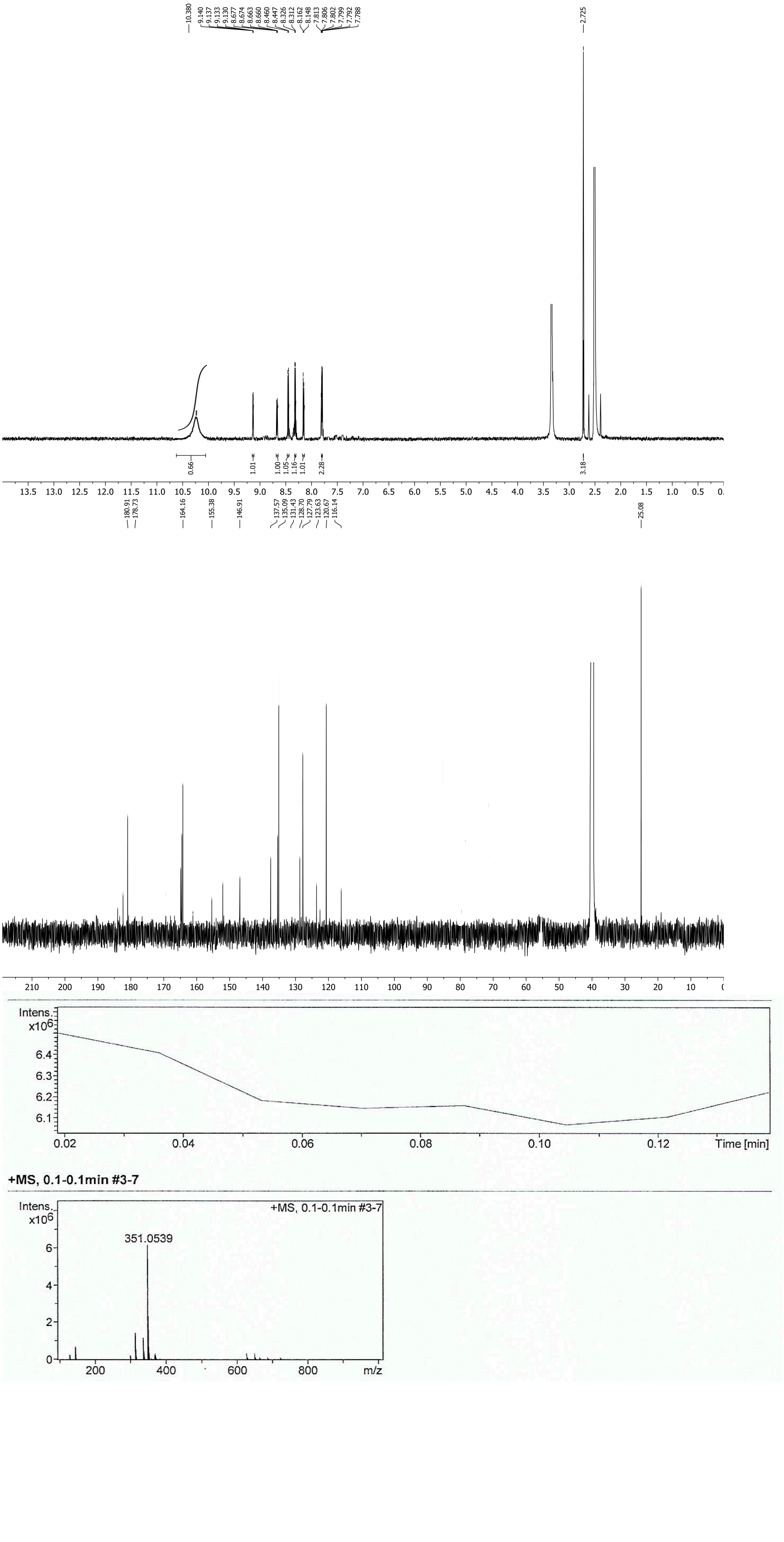

Figure S9: Spectra of 6-chloro-2-methyl-7-[(2-methylquinolin-8-yl)oxy]quinoline-5,8-dione (**12b**)

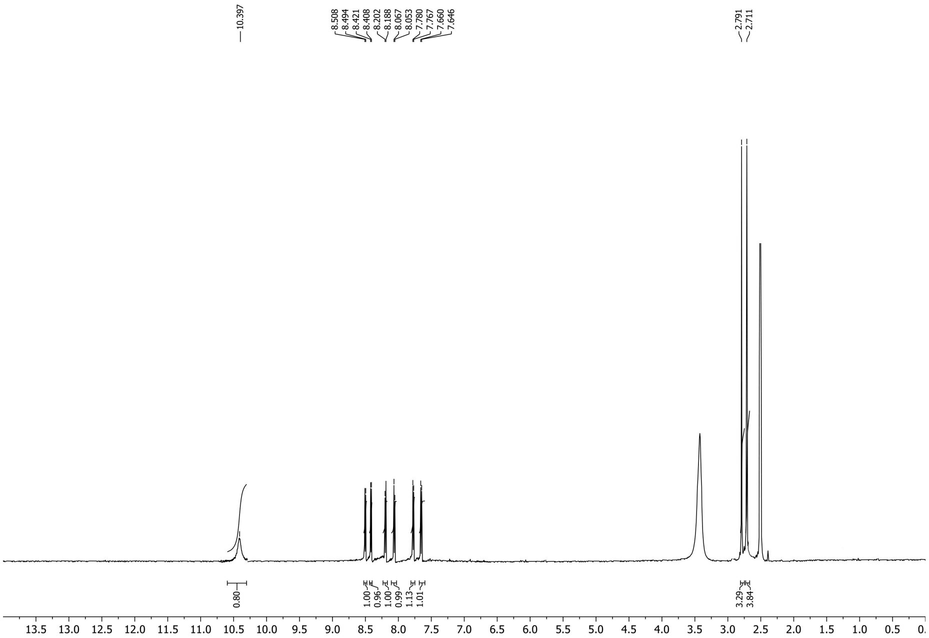

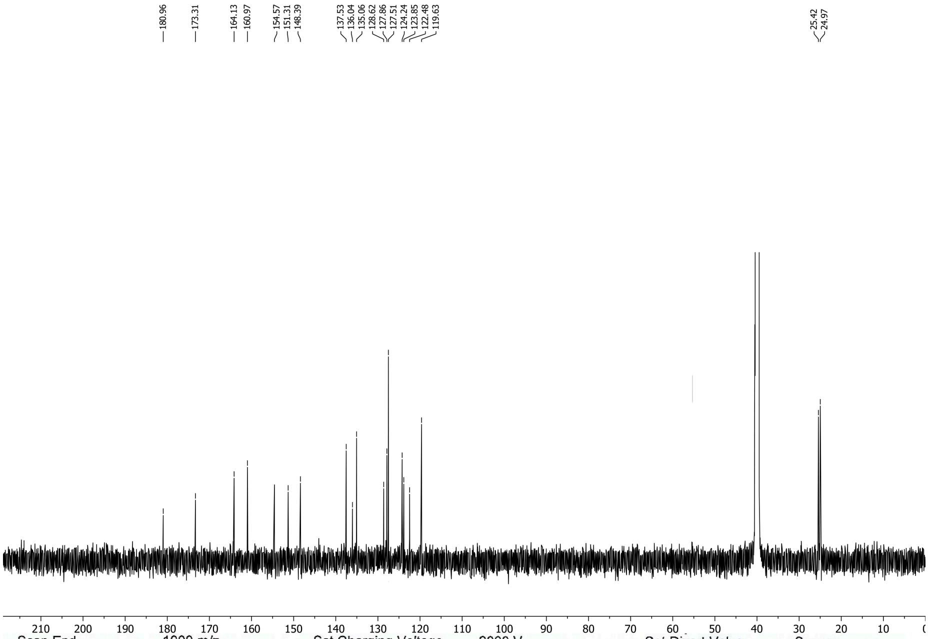

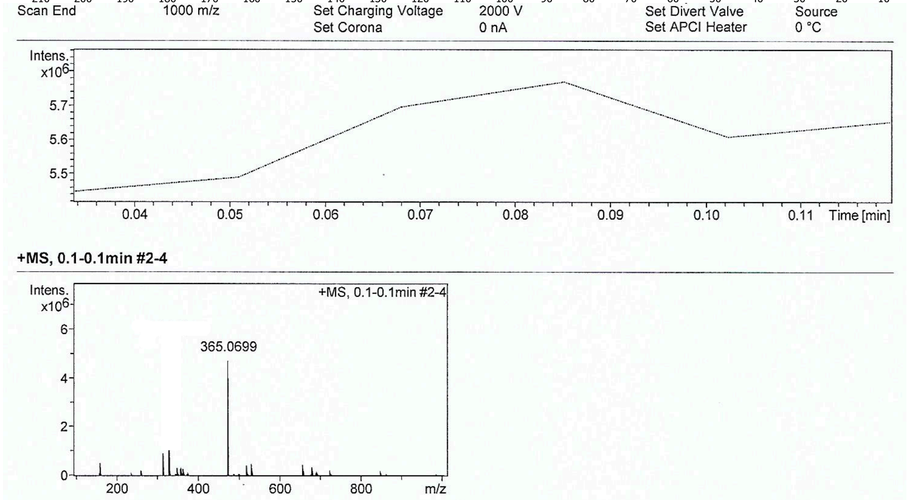

Figure S10: Spectra of 8-[(6-chloro-2-methyl-5,8-dioxo-5,8-dihydroquinolin-7-yl)oxy]quinoline-2-carbaldehyde (**12c**).

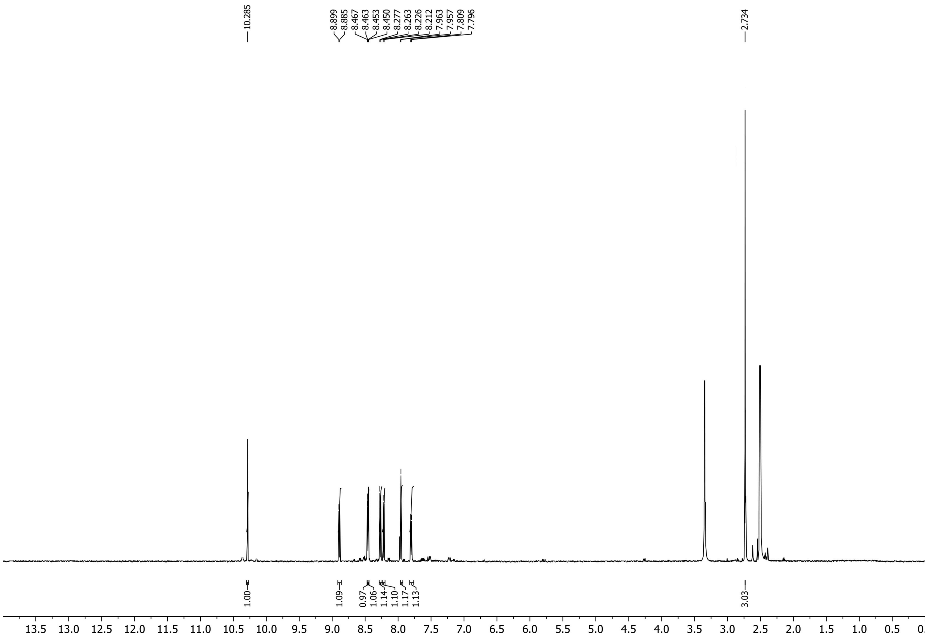

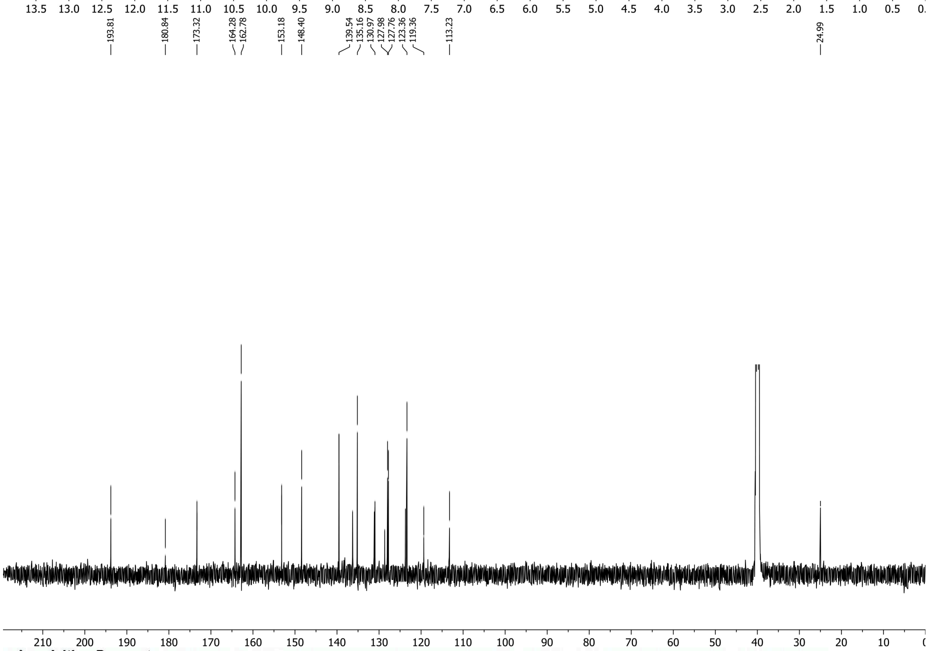

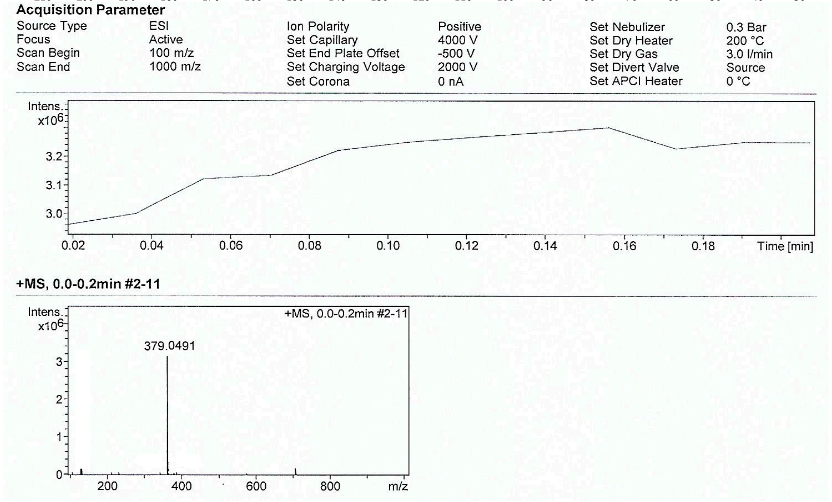

Figure S11: Spectra of 6-chloro-2-methyl-7-[(2-chloroquinolin-8-yl)oxy]quinoline-5,8-dione (**12d**).

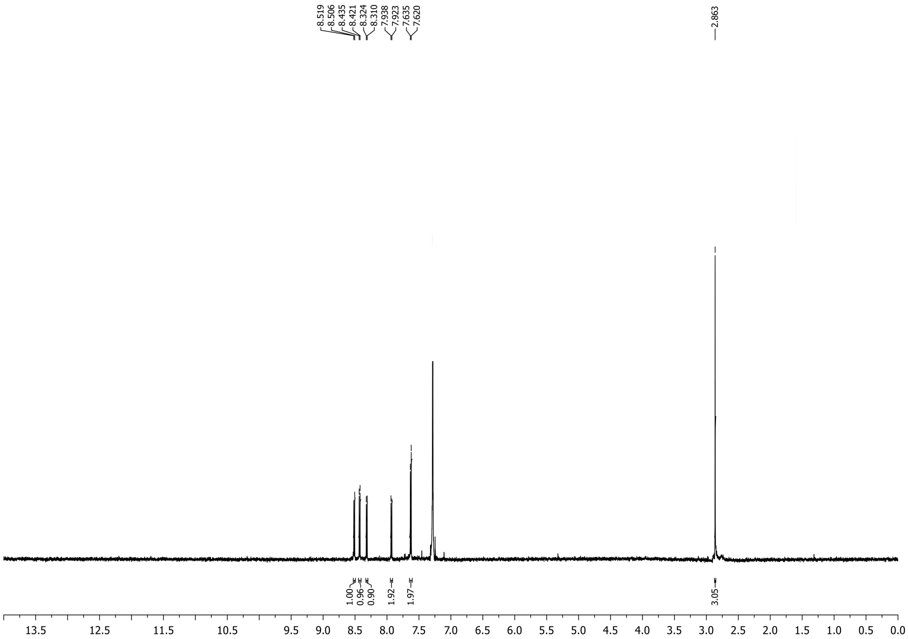

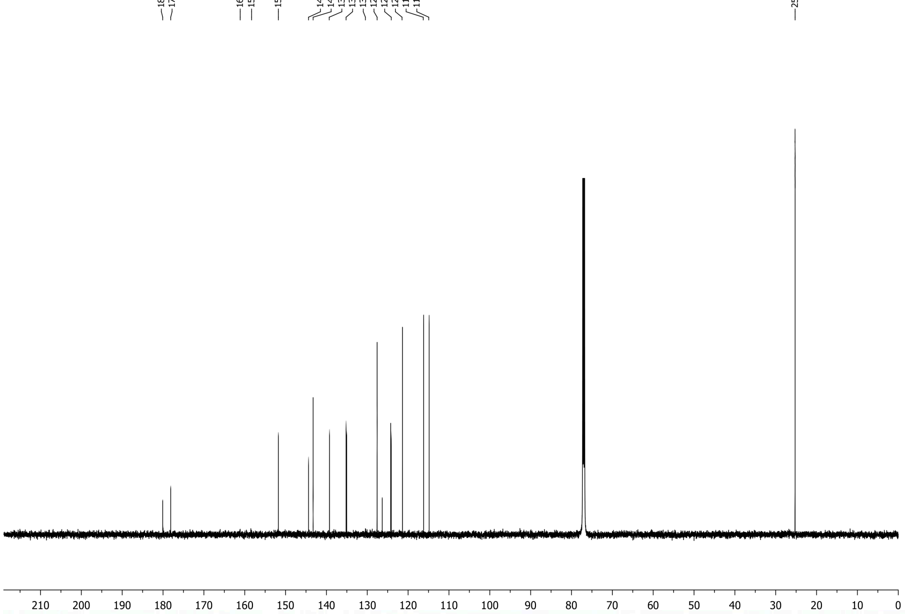

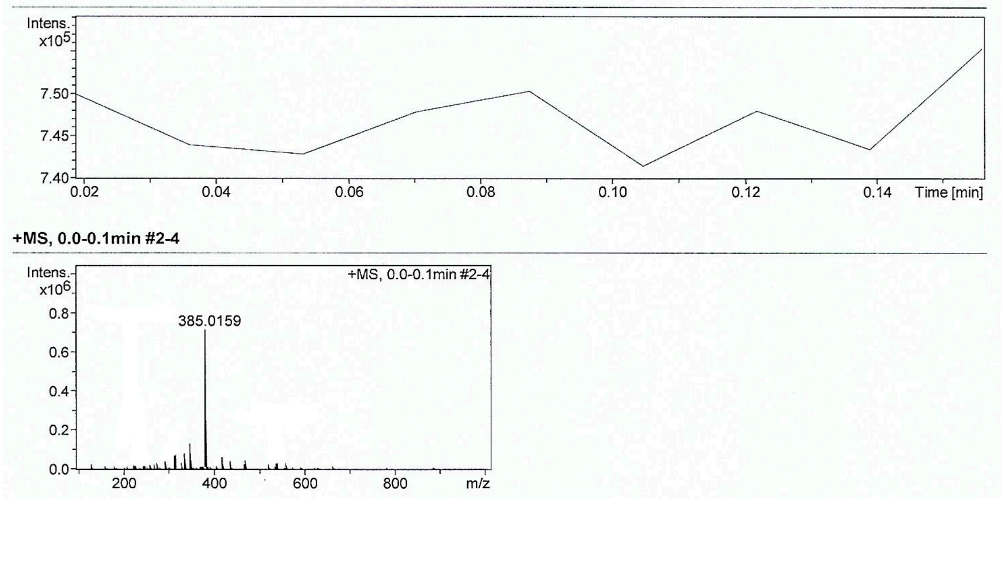

Figure S12: Spectra of 6-chloro-2-methyl-7-{[2-(pyrrolidin-1-yl)quinolin-8-yl]oxy}quinoline-5,8-dione (**12e**).

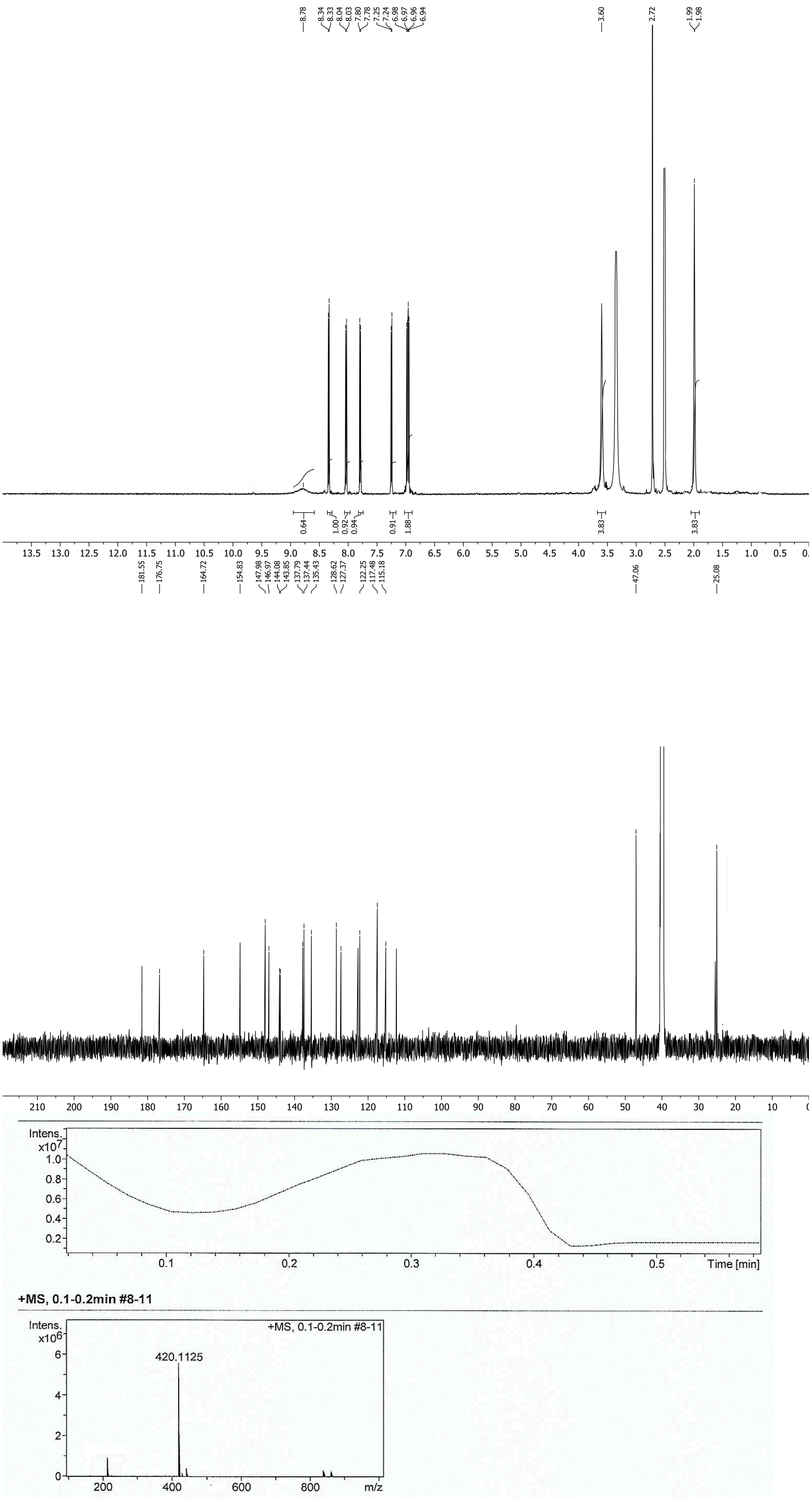

Figure S13: Spectra of 6-chloro-2-methyl-7-{[2-(morpholin-4-yl)quinolin-8-yl]oxy}quinoline-5,8-dione (**12f**).

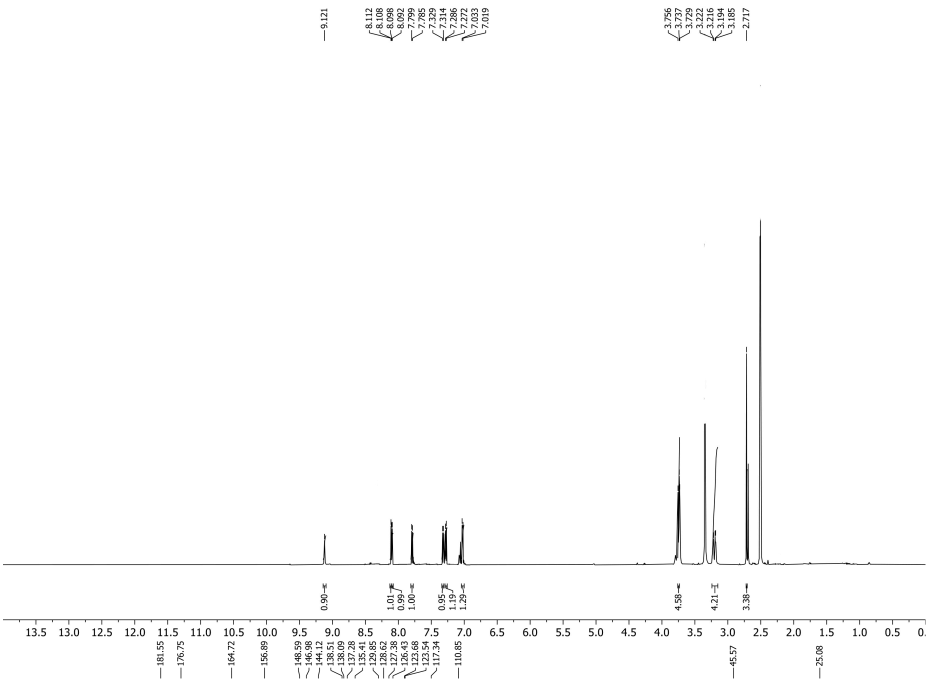

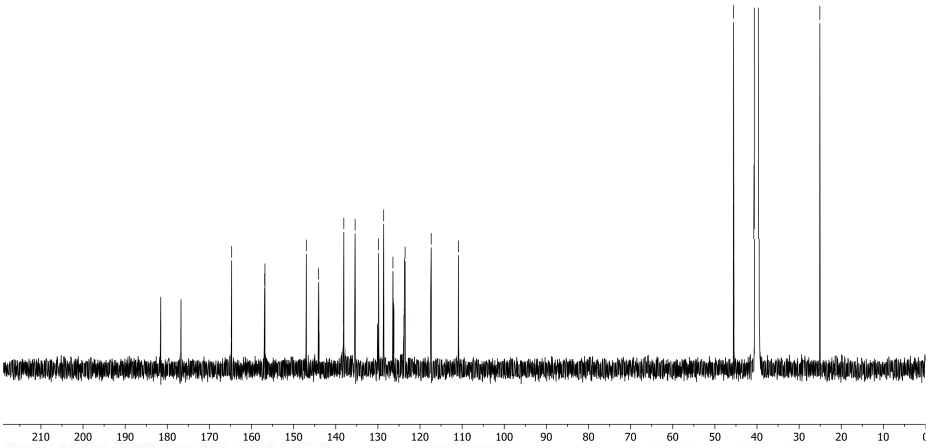

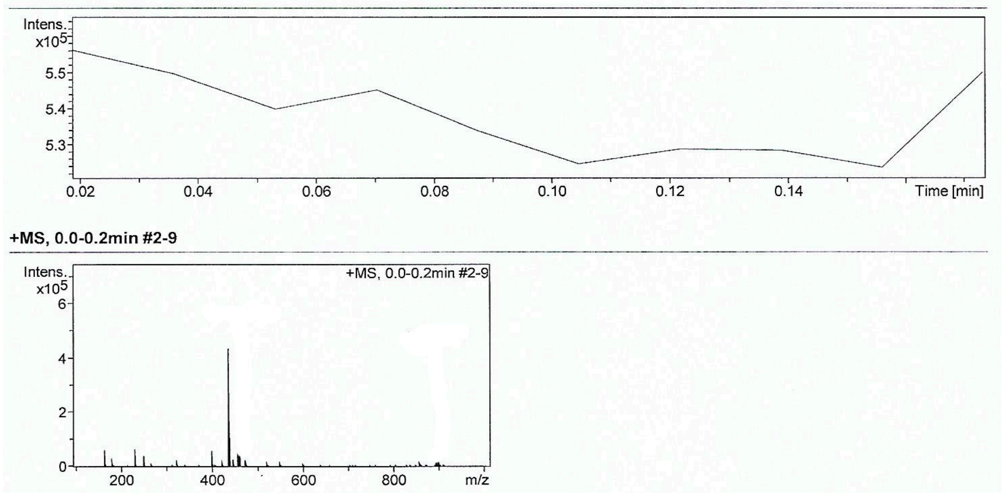

Figure S14: Spectra of 6-chloro-7-(quinolin-8-yloxy)isoquinoline-5,8-dione (**13a**).

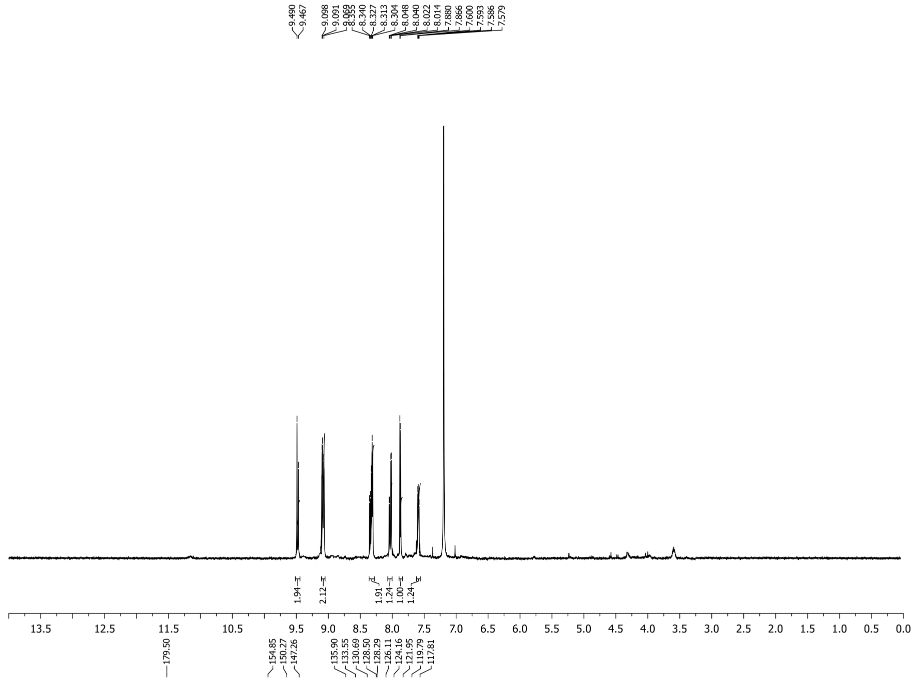

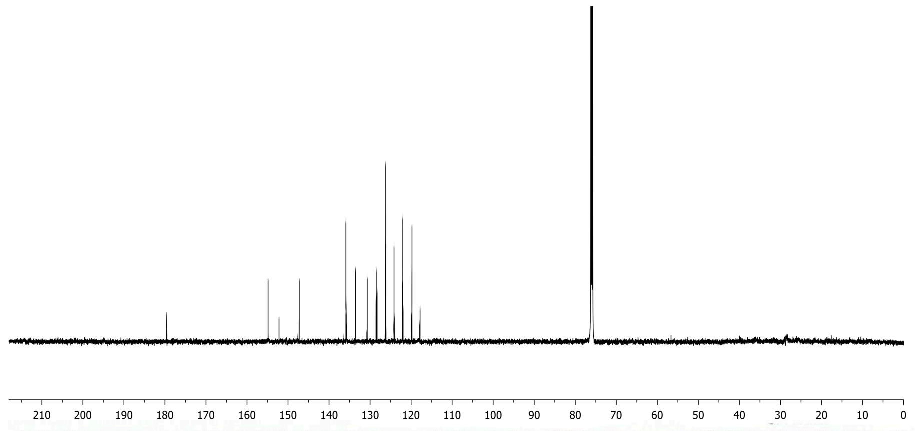

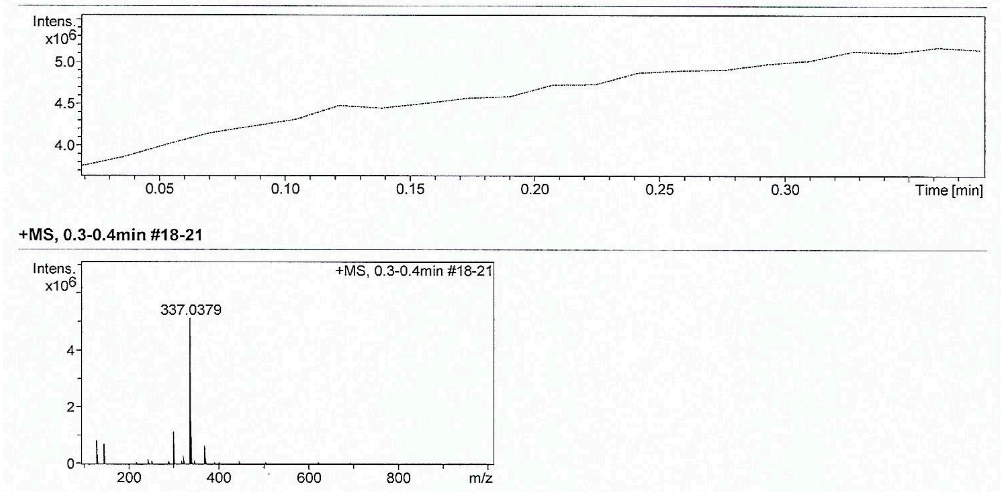

Figure S15: Spectra of 6-chloro-7-[(2-methylquinolin-8-yl)oxy]isoquinoline-5,8-dione (**13b**)

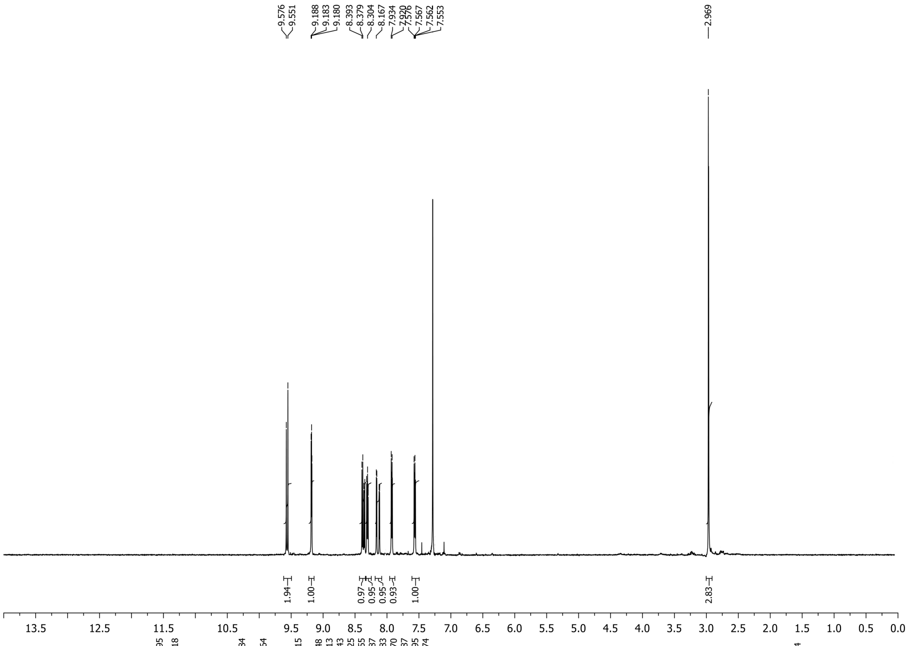

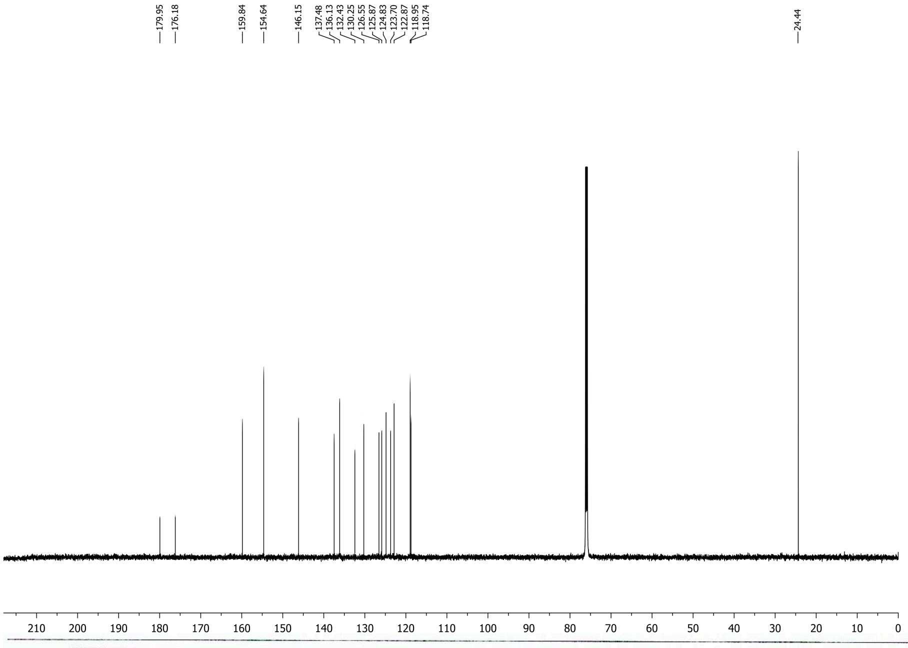

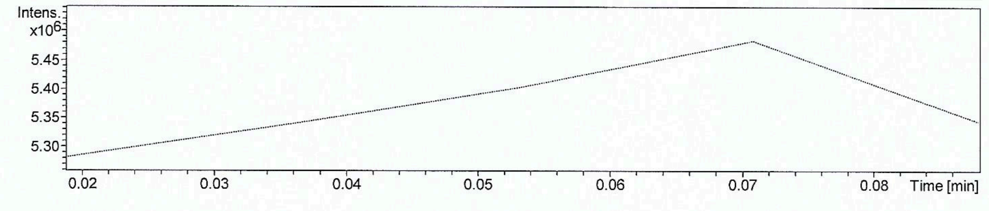

+MS, 0.0-0.1min #2-3

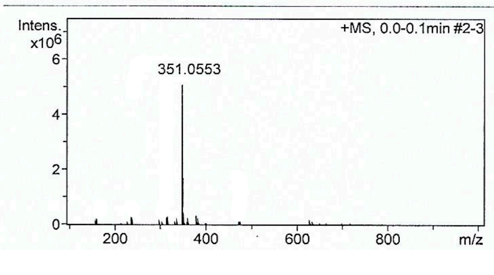

Figure S16: Spectra of 8-[(6-chloro-5,8-dioxo-5,8-dihydroquinolin-7-yl)oxy]isoquinoline-2-carbaldehyde (**13c**).

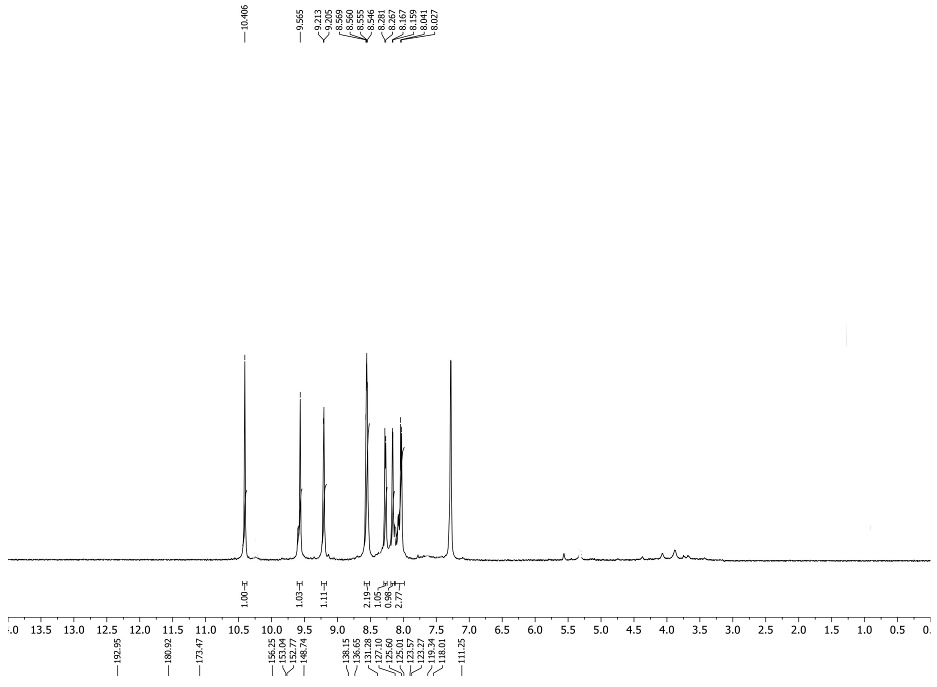

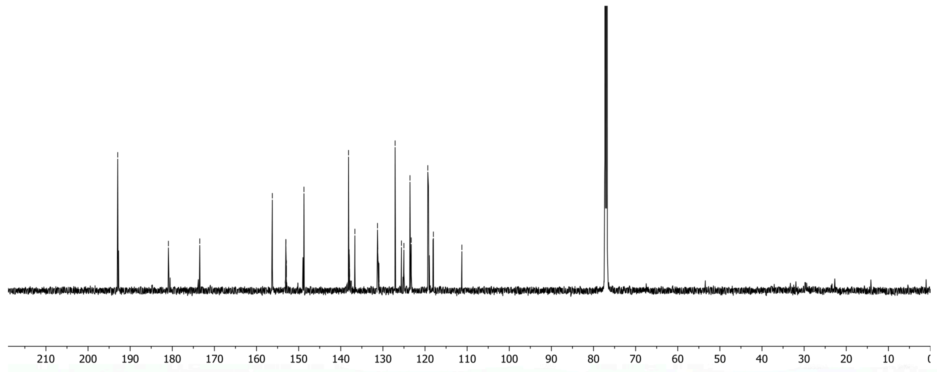

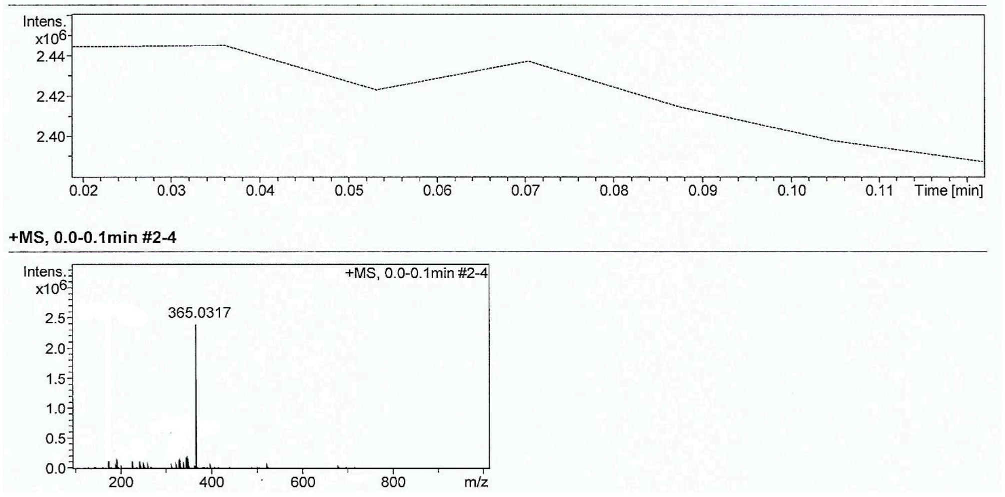

Figure S17: Spectra of 6-chloro-7-[(2-chloroquinolin-8-yl)oxy]isoquinoline-5,8-dione (**13d**).

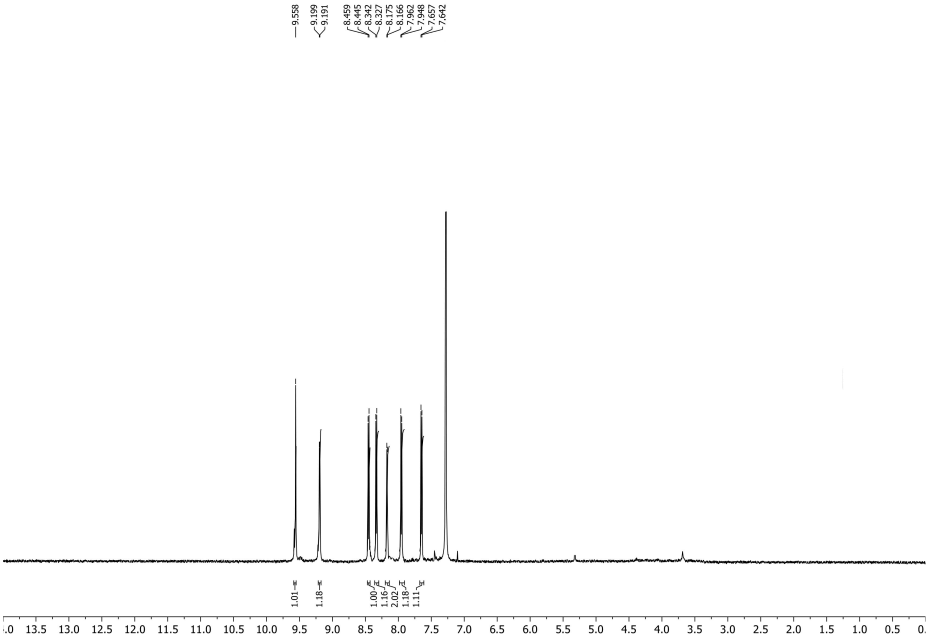

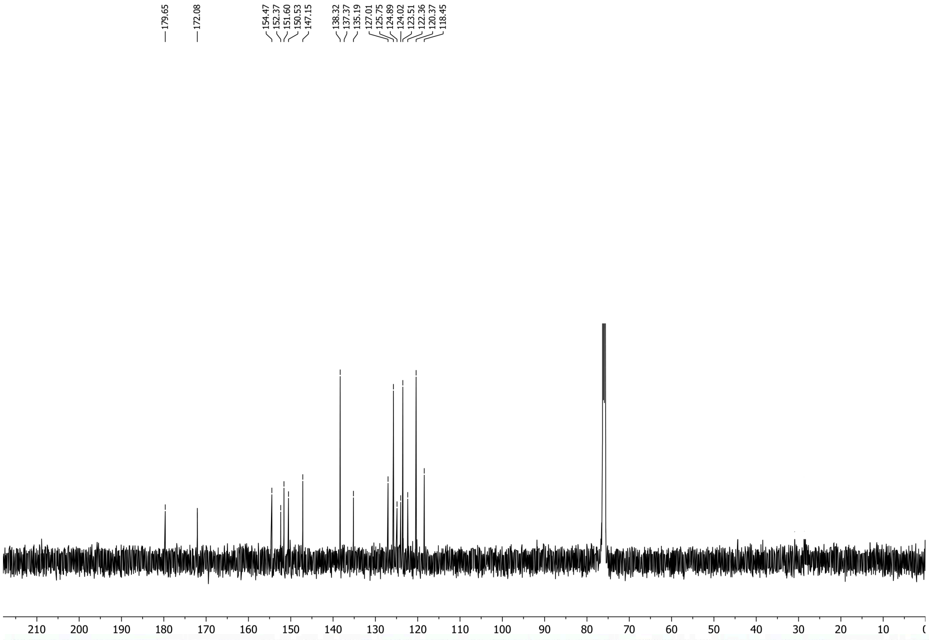

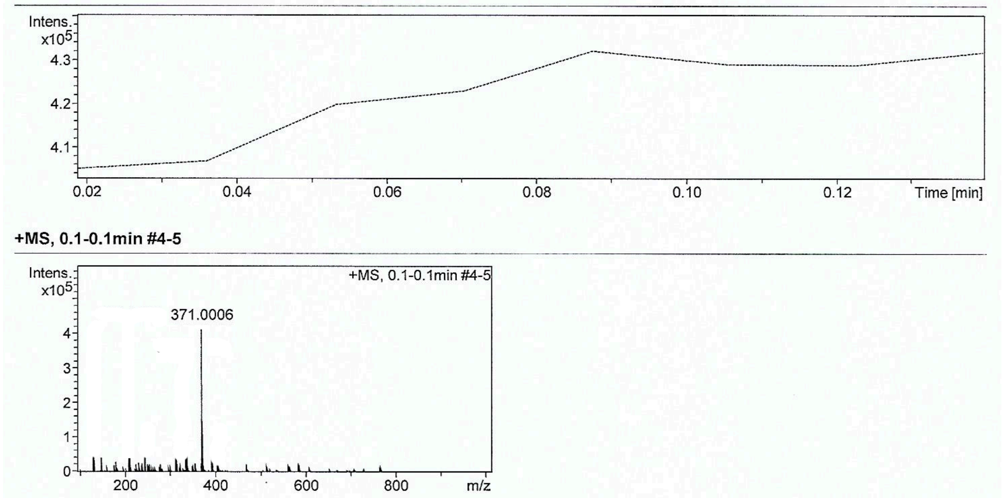

Figure S18: Spectra of 6-chloro-7-{[2-(pyrrolidin-1-yl)quinolin-8-yl]oxy}isoquinoline-5,8-dione (**13e**).

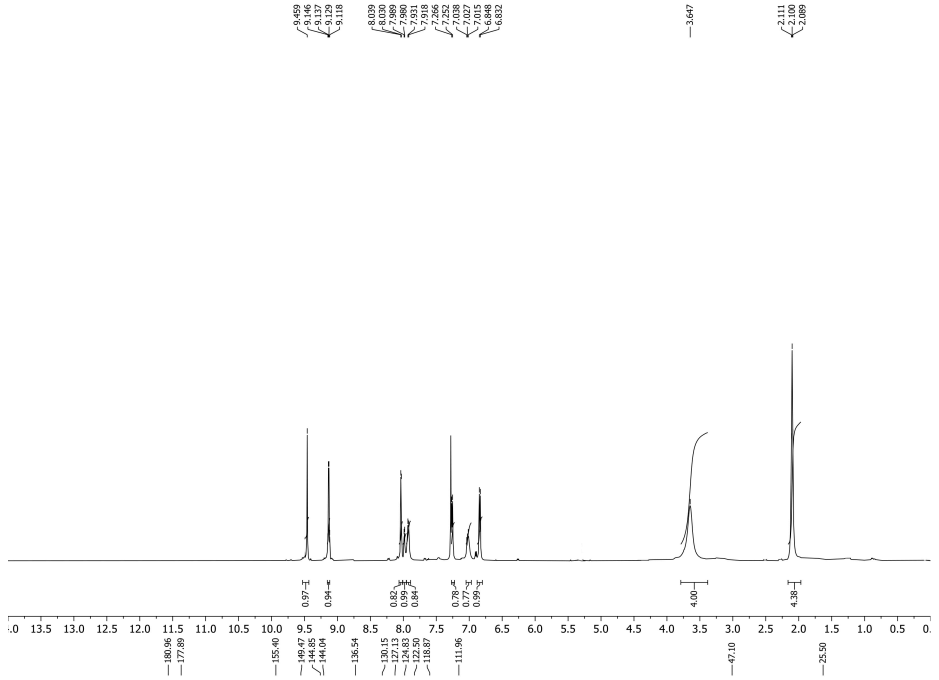

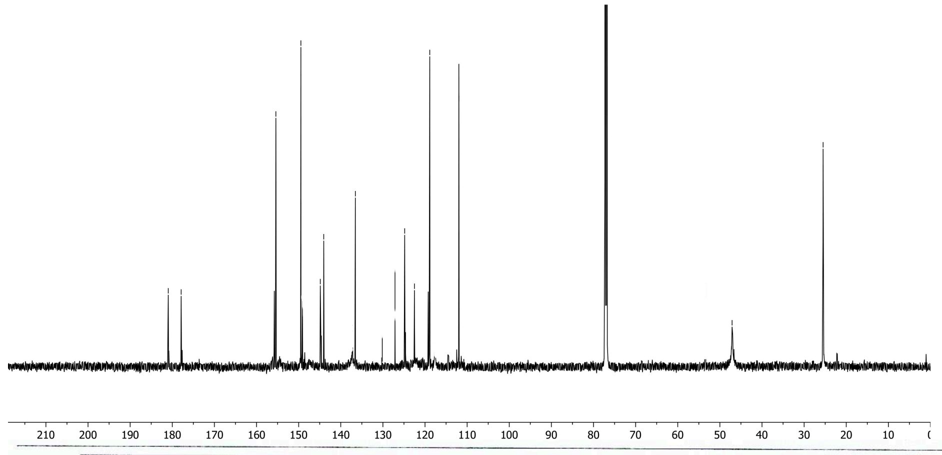

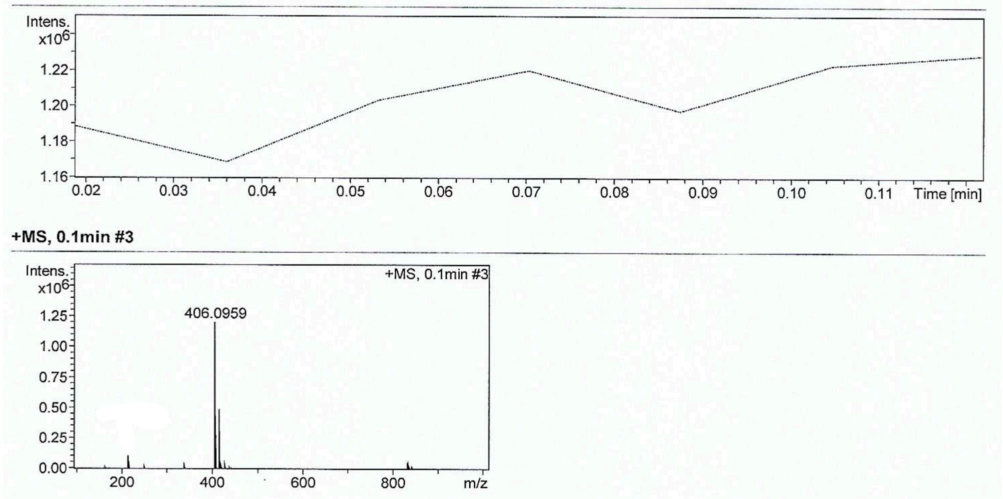

Figure S19: Spectra of 6-chloro-7-{[2-(morpholin-4-yl)quinolin-8-yl]oxy}isoquinoline-5,8-dione (**13f**).

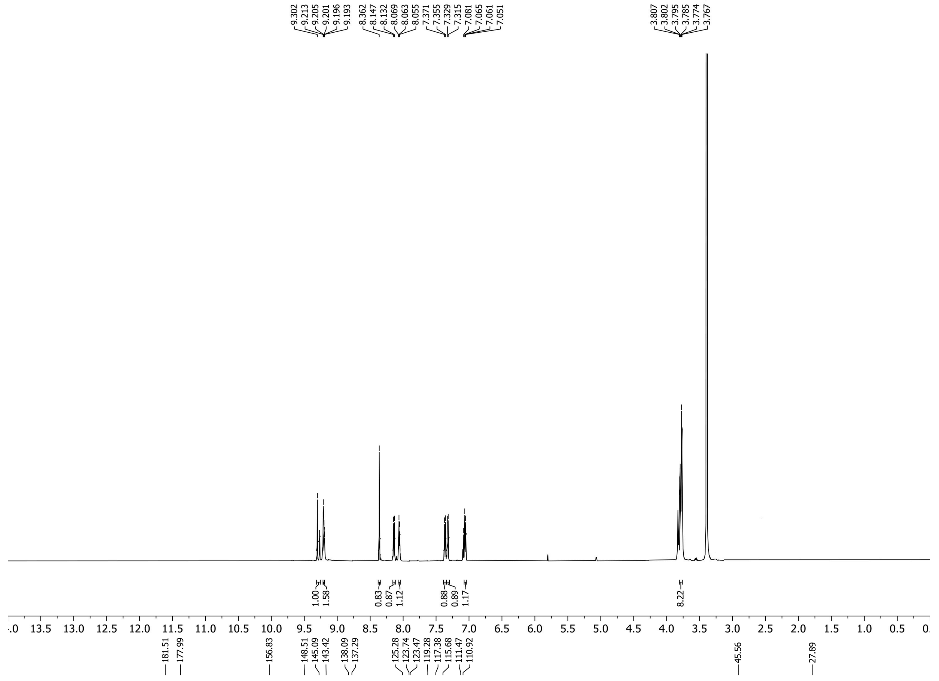

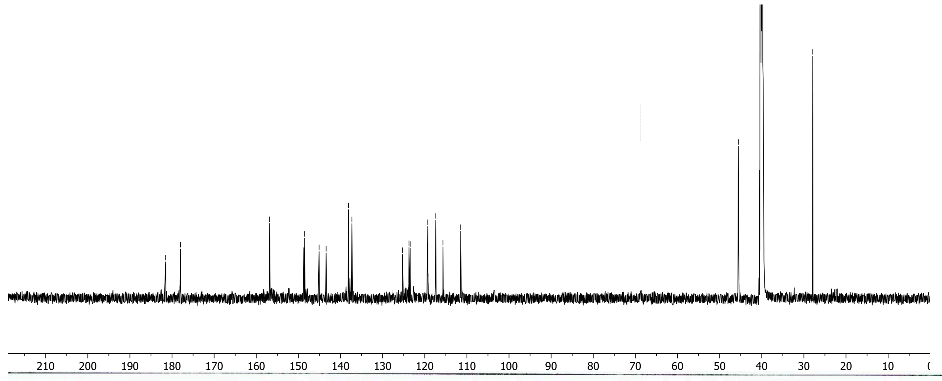

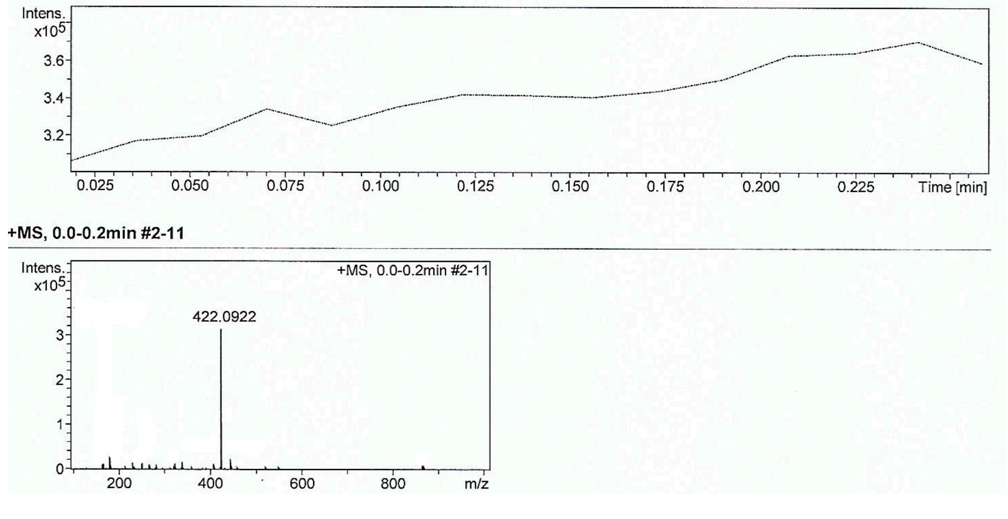

Figure S20: Spectra of 2-chloro-3-[(quinolin-8-yl)oxy]naphthalene-1,4-dione (**14a**).

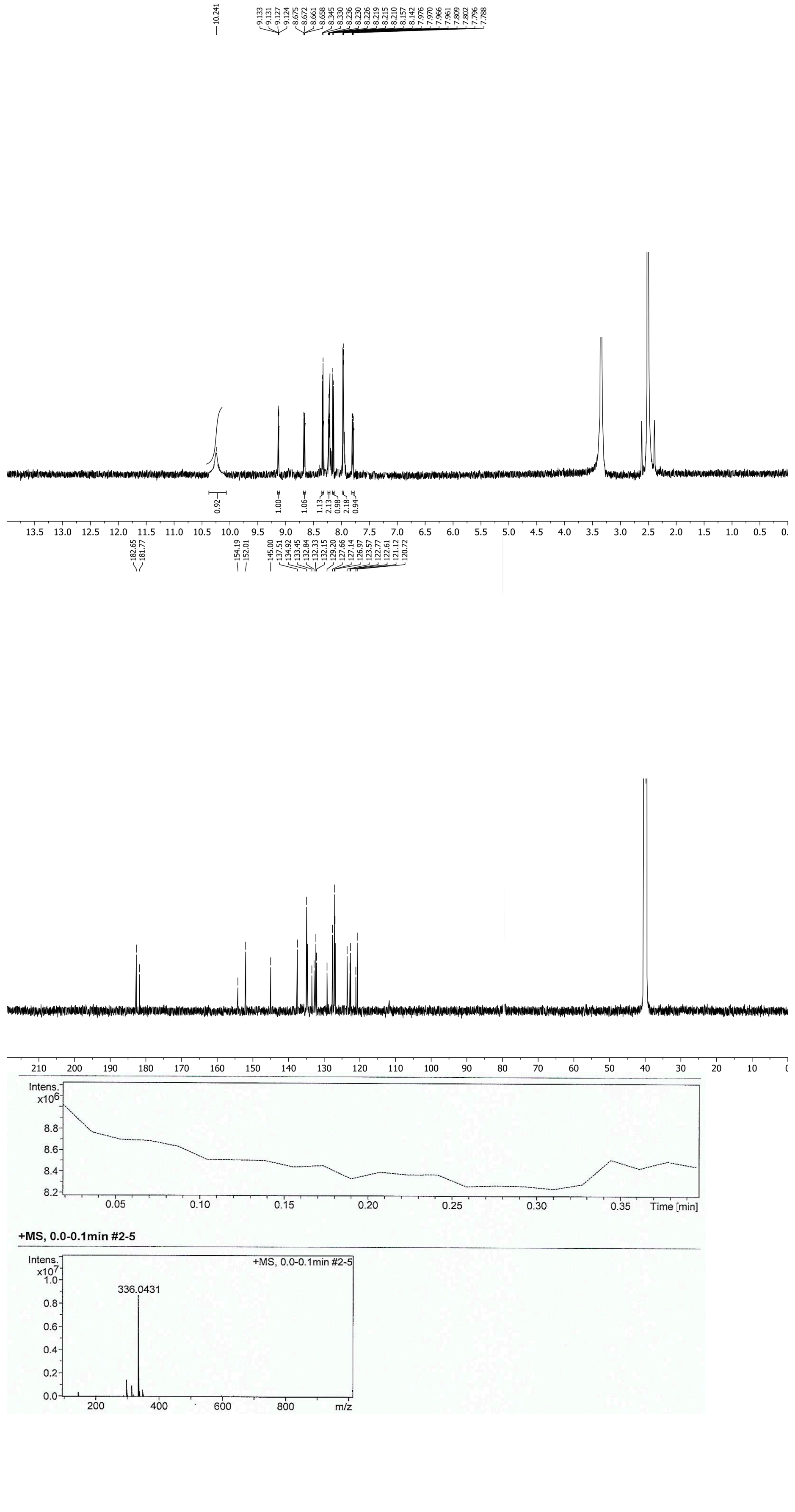

Figure S21: Spectra of 2-chloro-3-[(2-methylquinolin-8-yl)oxy]naphthalene-1,4-dione (**14b**).

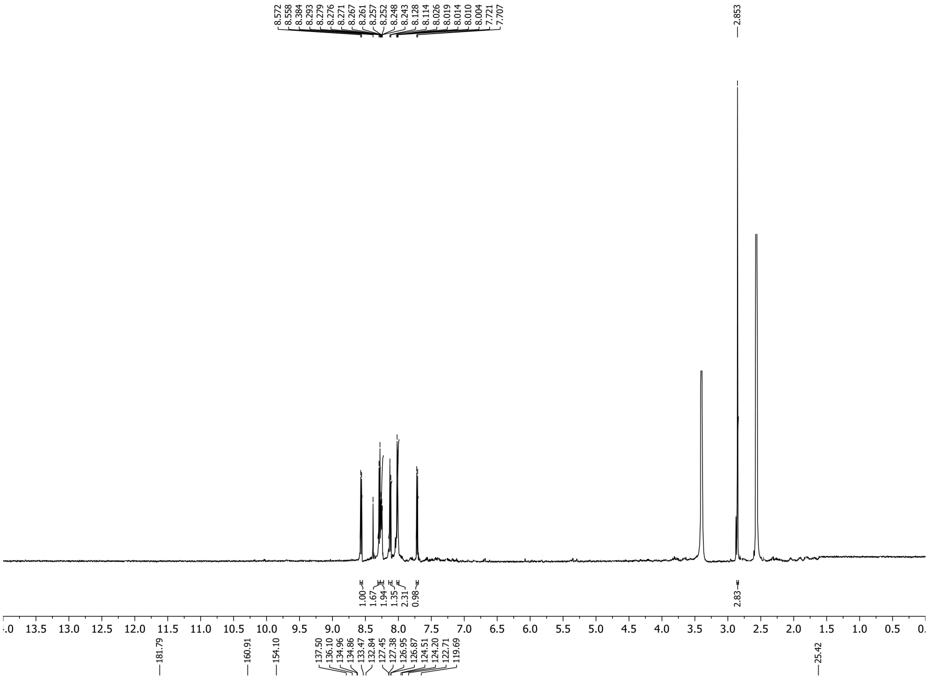

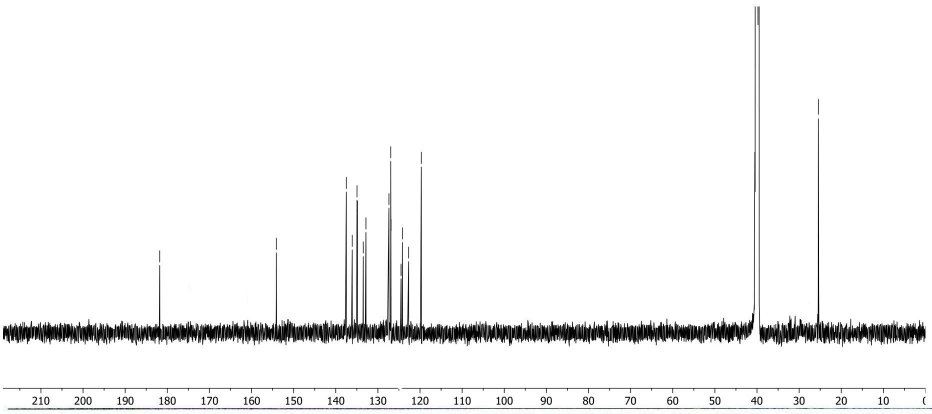

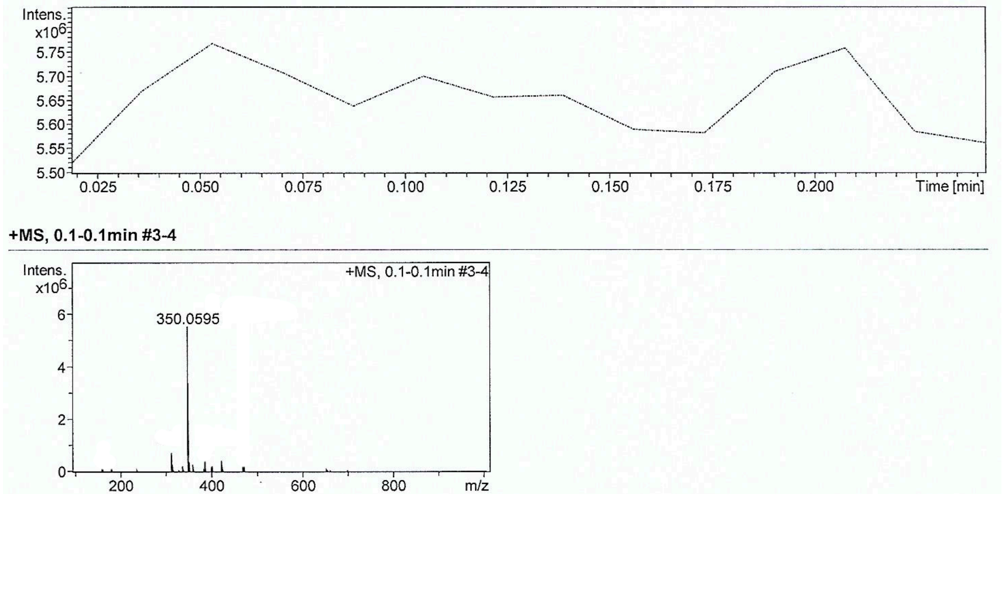

Figure S22: Spectra of 8-[(3-chloro-1,4-dioxo-1,4-dihydronaphthalen-2-yl)oxy]quinoline-2-carbaldehyde (**14c**).

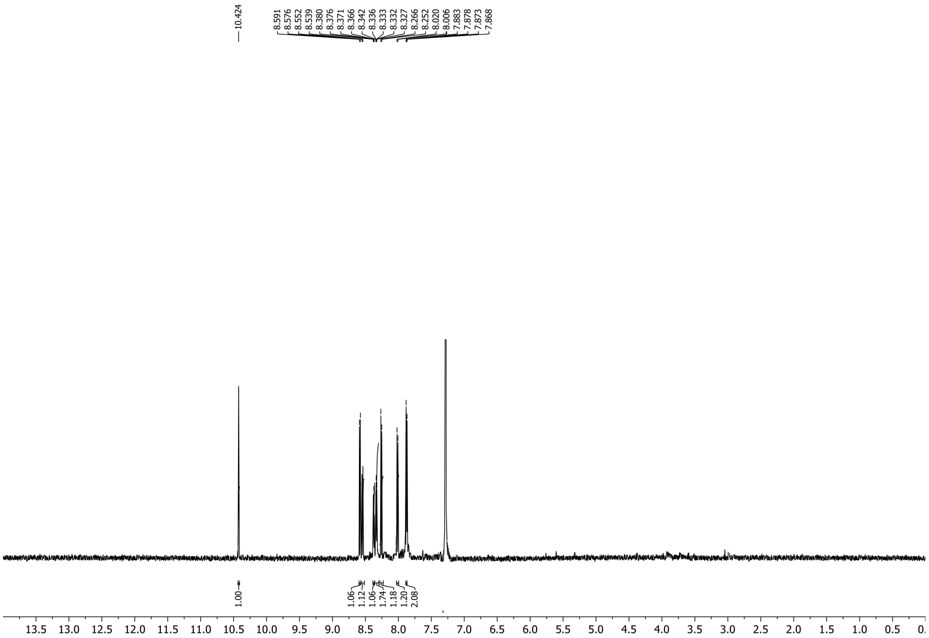

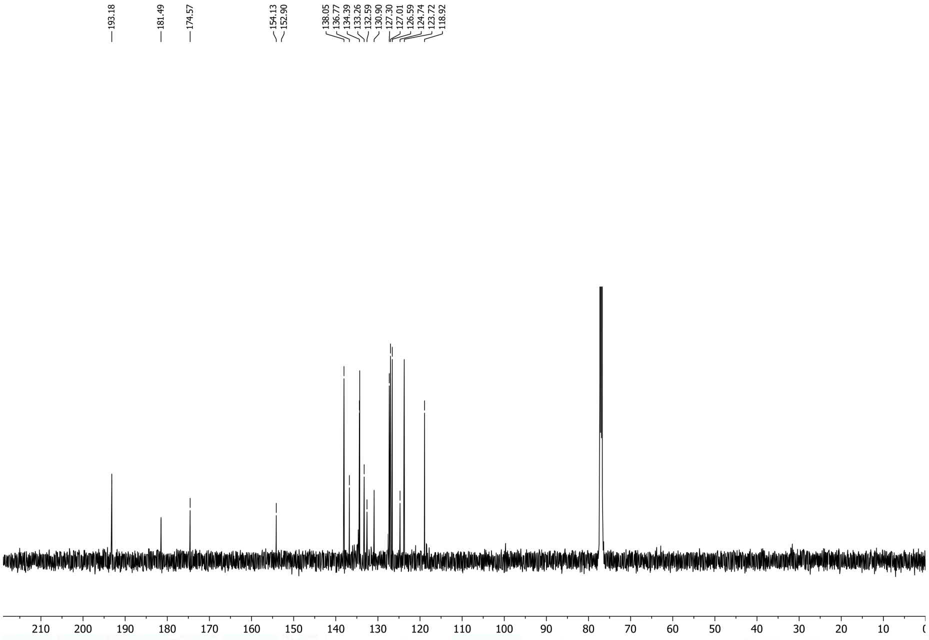

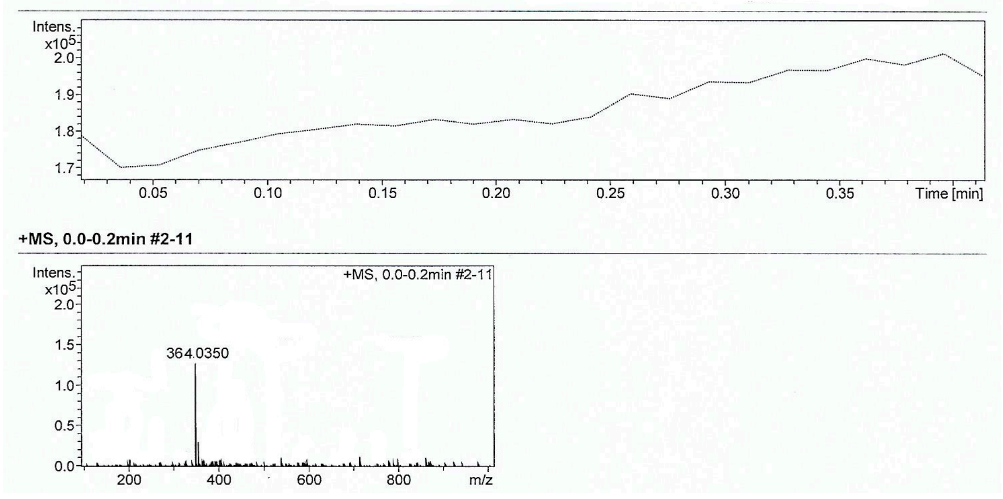

Figure S23: Spectra of 2-chloro-3-[(2-chloroquinolin-8-yl)oxy]naphthalene-1,4-dione (**14d**).

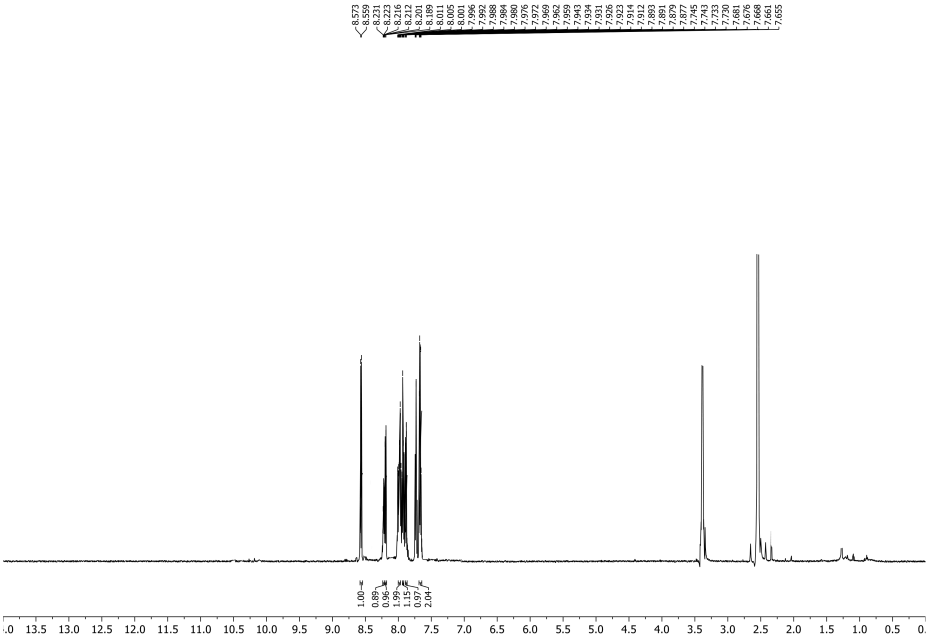

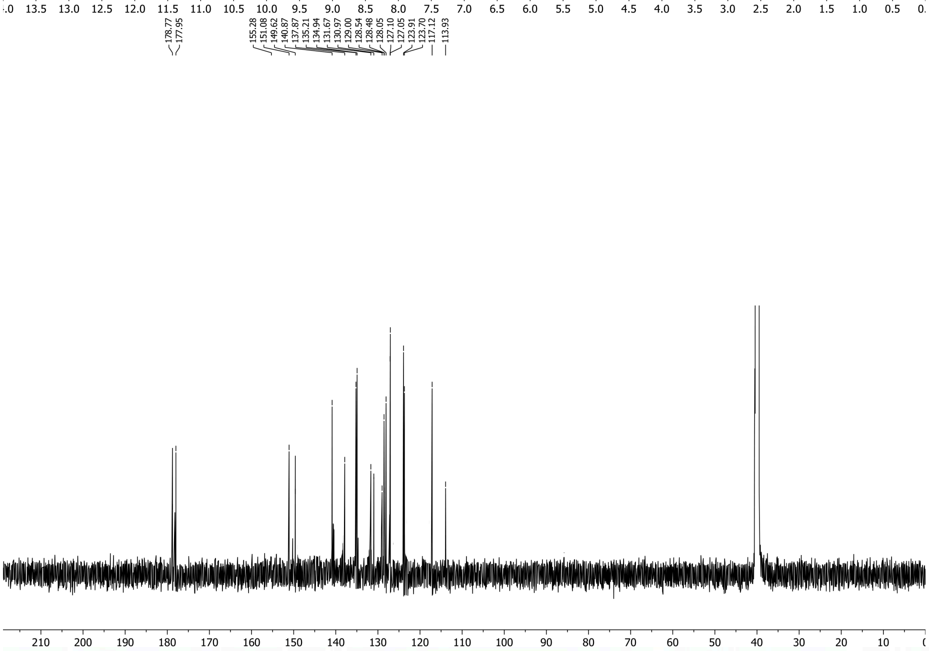

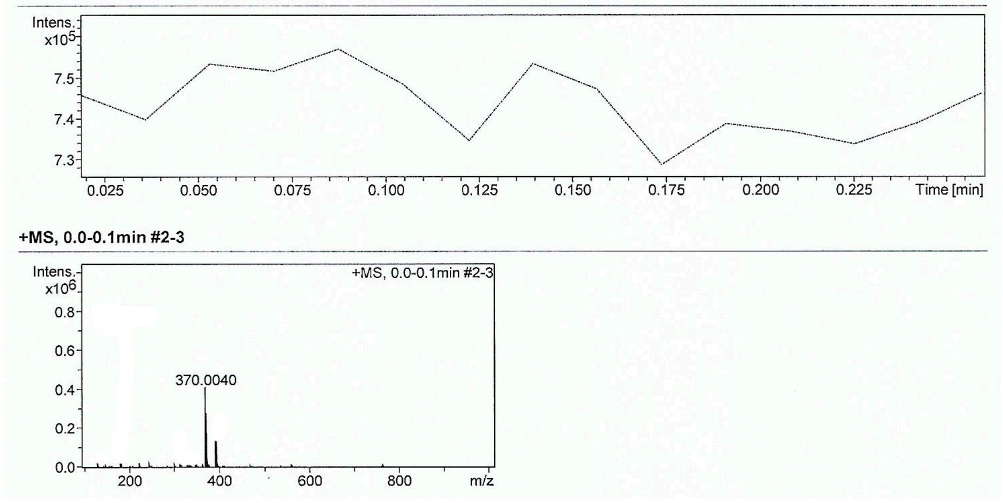

Figure S24: Spectra of 2-chloro-3-{[2-(pyrrolidin-1-yl)quinolin-8-yl]oxy}naphthalene-1,4-dione (**14e**).

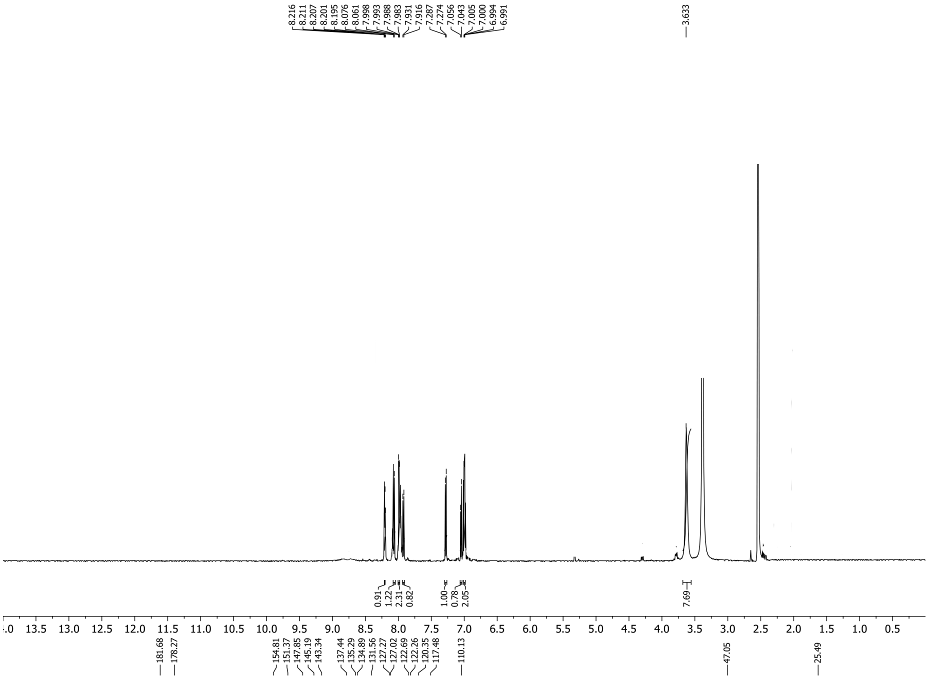

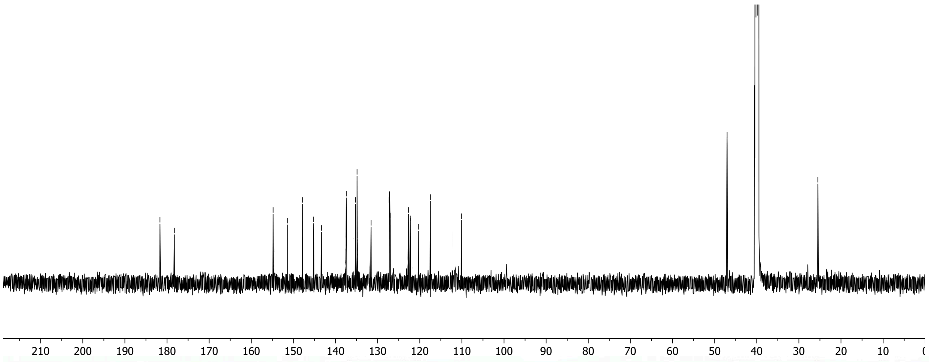

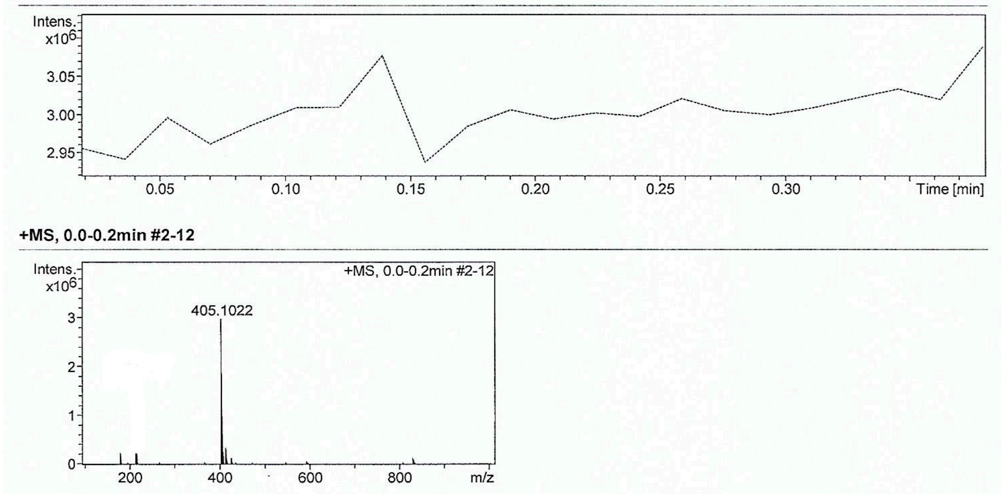

Figure S25: Spectra of 2-chloro-3-{[2-(morpholin-4-yl)quinolin-8-yl]oxy}naphthalene-1,4-dione (**14f**).

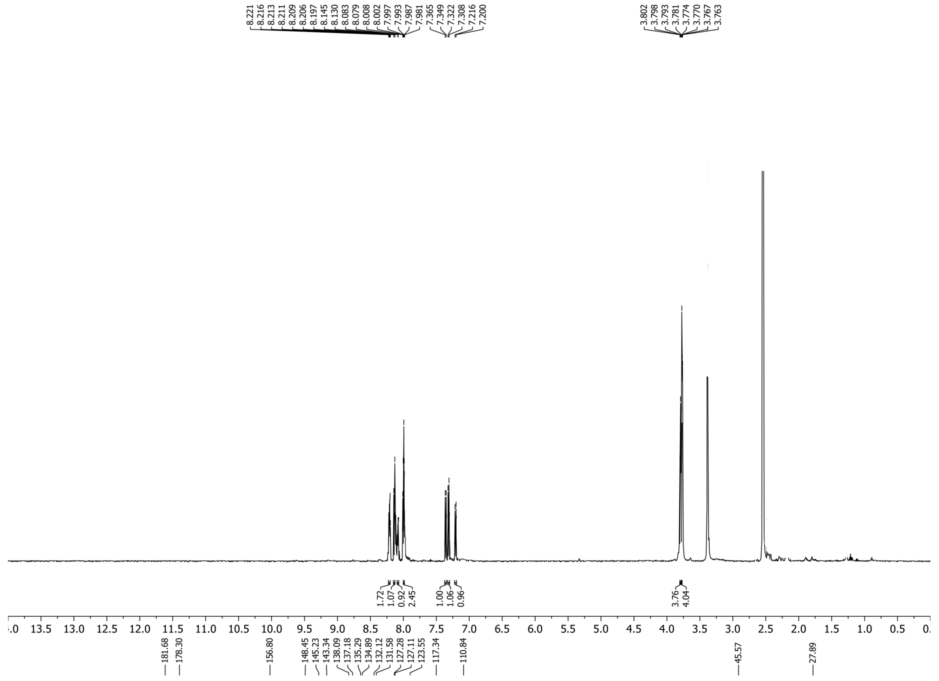

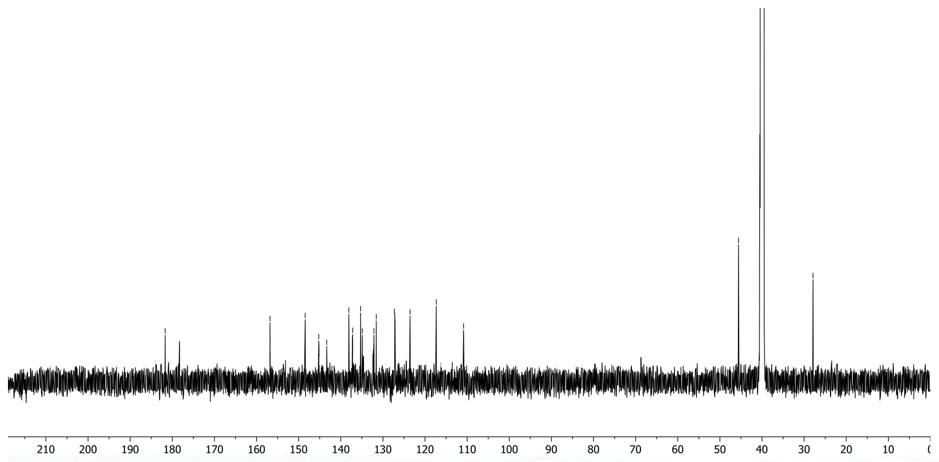

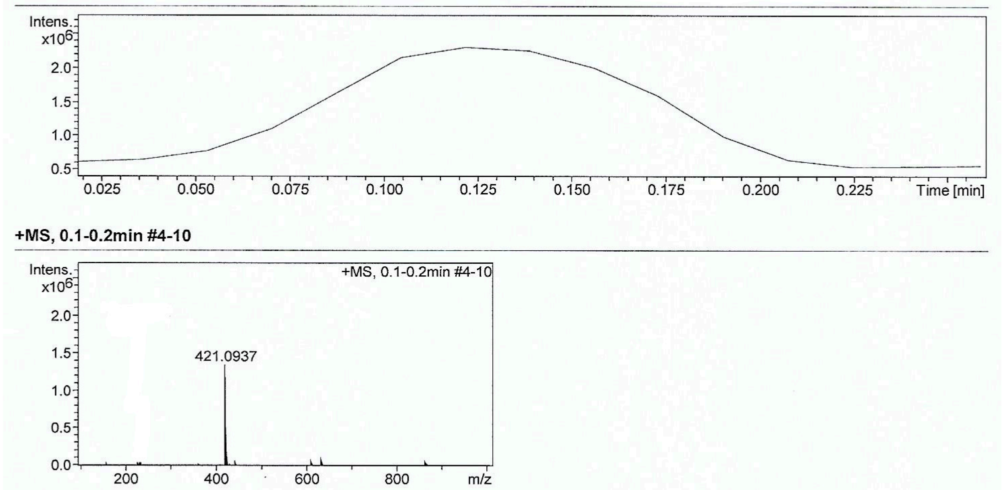

Supplement: Supplementary file 1 [file molecules-27-06206-s001.zip › molecules-1883018-supplementary.pdf]
